# *Saccharothrix camelliae* sp. nov., isolated from rhizosphere soil of *Camellia oleifera* Abel and proposal of *Saccharothrixyanglingensis* as a later heterotypic synonym of *Saccharothrix longispora*

**DOI:** 10.3389/fmicb.2026.1716500

**Published:** 2026-02-27

**Authors:** Ting Tang, Hang Jia, Yuhuan Cao, Jia Deng, Mingjun Ke, Ping Mo, Kaiqin Li, Jian Gao

**Affiliations:** 1 Key Laboratory of Agricultural Products Processing and Food Safety in Hunan Higher Education, Hunan Provincial Engineering Research Center for Fresh Wet Rice Noodles, Science and Technology Innovation Team for Efficient Agricultural Production and Deep Processing at General University in Hunan Province, Changde Key Innovation Team for Wetland Biology and Environmental Ecology, College of Life and Environmental Sciences, Hunan University of Arts and Science, Changde, Hunan, China; 2 College of Furong, Hunan University of Arts and Science, Changde, Hunan, China; 3 College of Synthetic Biology Industry, Hunan University of Arts and Science, Changde, China; 4 Hunan University of Science and Technology, Xiangtan, China

**Keywords:** *Camellia oleifera*, heterotypic synonym, novel species, polyphasic taxonomy, *Saccharothrix camelliae*

## Abstract

A novel actinobacterial strain, designated HUAS TT1^T^, was isolated from the rhizosphere soil of *Camellia oleifera* collected from Changde City, Hunan Province, China. Sequence analysis of the full-length 16S rRNA gene revealed that strain HUAS TT1^T^ belonged to the genus *Saccharothrix* and shared the highest sequence similarities with *Saccharothrix yanglingensis* Hhs.015^T^ (99.30%), *Saccharothrix carnea* NEAU-yn17^T^ (98.89%), *Saccharothrix hoggarensis* SA181^T^ (98.89%), *Saccharothrix saharensis* SA152^T^ (98.82%), and *Saccharothrix longispora* NRRL B-16116^T^ (98.75%). Phylogenetic analysis based on 16S rRNA gene sequences indicated that strain HUAS TT1^T^ was related to *Saccharothrix yanglingensis* Hhs.015^T^ and *Saccharothrix longispora* NRRL B-16116^T^. Phylogenetic analysis based on whole-genome sequences and 120 core gene sequences showed that the evolutionary neighbors of strain HUAS TT1^T^ were *Saccharothrix hoggarensis* SA181^T^ and *Saccharothrix saharensis* SA152^T^. However, the ANIb/m and dDDH values between the whole-genome sequences of strain HUAS TT1^T^ and *Saccharothrix hoggarensis* SA181^T^/*Saccharothrix saharensis* SA152^T^ were 85.82%/88.80 and 32.90%, 86.42%/89.43, and 34.40%, respectively, well below the 95–96 and 70% cut-off points recommended for delineating species, suggesting that strain HUAS TT1^T^ is a novel *Saccharothrix* species. This result was further verified by the phenotypic and chemotaxonomic differences between them. Therefore, according to the above data, strain HUAS TT1^T^ represents a novel species of the genus *Saccharothrix*, for which the name *Saccharothrix camelliae* sp. nov. is proposed. The type strain used was HUAS TT1^T^ (=MCCC 1K09364^T^ = JCM 37292^T^). In addition, the phenotypic, chemotaxonomic, and genotypic characteristics, as well as the phylogenetic information, indicated that *S. yanglingensis* CGMCC 4.5627^T^ and *S. longispora* CGMCC 4.1357^T^ belong to the same species. Therefore, on the basis of these results and Rule 42 of the Bacteriological Code, we propose that *Saccharothrix yanglingensis* is a later heterotypic synonym of *Saccharothrix longispora*, for which an emended description is given.

## 1 Introduction

In recent years, the extensive use of antibiotics in both medical treatment and livestock breeding has led to a continuous increase in bacterial resistance, posing a grave threat to human life and health. To address this issue, there is an urgent need to discover drugs with novel structures and activities (Brown and Wright, 2016; Tacconelli et al., 2018; Kim, 2020). Natural products have assumed an increasingly pivotal role in the course of drug discovery owing to the diversity of their chemical structures and remarkable biological activities. Recent studies have shown that approximately 46% of 1,881 drugs approved for marketing between 1981 and 2019 were natural products and their analogs (Newman and Cragg, 2020). Actinomycetes represent a significant reservoir of natural products, accounting for approximately two-thirds of all bioactive metabolites derived from microorganisms. Among these, streptomycetes are the predominant contributors, producing approximately 76% of these compounds (Bérdy, 2012). However, the efficiency of novel antibiotics produced by *Streptomyces* has declined in recent years. An increasing number of reports have indicated that rare actinomycetes can serve as “new reservoirs” of natural products, holding great potential for yielding novel-structured bioactive products (Tiwari and Gupta, 2012; Zhang et al., 2019; Ding et al., 2019; Al-fadhli et al., 2022; Parra et al., 2023).

As a typical rare actinomycete, *Saccharothrix*, which belongs to the family *Pseudonocardiaceae*, was first described by Labeda et al. (1984) and includes over 20 species with validated published names^1^ at the time of writing. According to the Natural Products Atlas database,^2^ there are more than 70 compounds that have been isolated from the genus *Saccharothrix*, such as cyanogrisides I–J, iso-hexanoyl-pyrrothine (a dithiolopyrrolone antibiotic), two new angucyclines (saccharothrixmicines A–B), and ammocidins A–B (Kalinovskaya et al., 2010; Lahoum et al., 2019; Merrouche et al., 2019; Wei et al., 2024). It is important to note that proper classification of these organisms is a prerequisite for the systematic exploration of their biosynthetic potential.

During our search for novel actinobacteria that produce bioactive compounds, a novel *Saccharothrix* strain, designated HUAS TT1^T^, was isolated from the rhizosphere soil of *Camellia oleifera*. In this study, the taxonomic status of strain HUAS TT1^T^ was determined using a polyphasic taxonomic approach. Furthermore, in the course of identifying strain HUAS TT1^T^, it was found that *Saccharothrix yanglingensis* Hhs.015^T^ and *Saccharothrix longispora* JCM 3314^T^ could belong to the same species. Therefore, another purpose of this study is to re-evaluate the taxonomic relationship between these two species, *S. yanglingensis* and *S. longispora*.

## 2 Methods

### 2.1 Isolation and maintenance

Rhizosphere soil from *Camellia oleifera* Abel was collected from Changde City, Hunan Province, PR China (28°57′13.5650427259824″N, 111°26′3.37750246118412″E). Strain HUAS TT1^T^ was isolated using the dilution plate method. Modified Gause′s synthetic No.1 medium (Mo et al., 2018) was used to isolate strain HUAS TT1^T^ by adding a K_2_Cr_2_O_7_ solution. Gause′s synthetic No.1 medium (Atlas, 1993) was used to purify and preserve strain HUAS TT1^T^. The purified strain HUAS TT1^T^ was prepared in 30% glycerol at −80 °C or freeze-dried for long-term preservation. The type strains, *Saccharothrix hoggarensis* DSM 45457^T^ (=CCUG 60214^T^), *Saccharothrix saharensis* DSM 45456^T^ (=SA152^T^), *Saccharothrix yanglingensis* CGMCC 4.5627^T^ (=Hhs.015^T^), and *Saccharothrix longispora* CGMCC 4.1357^T^ (=JCM 3314^T^), were obtained from DSMZ (Deutsche Sammlung von Mikroorganismen und Zellkulturen, Germany) and CGMCC (China General Microbiological Culture Collection Center, Beijing, China), respectively. Strain HUAS TT1^T^ and the four above-mentioned reference strains were cultured under the same conditions for comparative analysis. Comparative experiments were performed in triplicate.

### 2.2 Morphological, cultural, and physiological characteristics

Cultures on Reasoner’s 2A agar (R2A medium; Reasoner and Geldreich, 1985), after incubation for 14 days at 28 °C using the insert method (Jiang and Ruan, 1982), were used for observation of morphological characteristics. The spore-chain, spore-shape, and spore-surface features of strain HUAS TT1^T^ were observed using a light microscope (NE620; Ningbo Yongxin Optics Co., Ltd., China) and a scanning electron microscope (FEIXL30; Phillip Electron Optics, now Thermo Fisher Scientific, USA). Gause′s synthetic No.1 medium, R2A medium, and ISP 2–7 agar media (Shirling and Gottlieb, 1966) were used to observe the cultural characteristics of strain HUAS TT1^T^ and the above-mentioned four reference strains after incubation for 21 days at 28 °C. The color of the aerial mycelium, substrate mycelium, and diffusible pigment was recorded using color standards and color nomenclature (Ridgway, 1912). Growth temperatures (4, 10, 15, 18, 25, 28, 32, 40, and 45 °C), growth pH (3, 4, 5, 6, 7, 8, 9, 10, and 11), and NaCl tolerance concentrations (0, 1, 2, 3, 4, 5, 6, and 7% w/v) were tested on ISP 2 liquid medium after incubation for 14 days at 28 °C. The liquefaction of gelatin, hydrolysis of starch, reduction of nitrate, and degradation of Tween 20, Tween 40, Tween 60, and Tween 80 were tested as described by Xu et al. (2007) and Ruan and Huang (2011). Biolog GEN III MicroPlates and the API ZYM system were used to determine the carbon and nitrogen source utilization and activities of constitutive enzymes in all tested strains. The assays were performed according to the manufacturer’s instructions. Comparative experiments were performed in triplicate.

### 2.3 Chemotaxonomic features

Strains HUAS TT1^T^, *S. hoggarensis* DSM 45457^T^, *S. saharensis* DSM 45456^T^, *S. yanglingensis* CGMCC 4.5627^T^, and *S. longispora* CGMCC 4.1357^T^ were cultivated in 120 mL tryptic soy broth in 250 mL shake flasks for 4 days at 28 °C and 130 rpm, and then the cells were collected for chemotaxonomic analysis. The fatty acids of the five strains were extracted, purified, methylated, and quantified using the Sherlock Microbial Identification system (MIDI system; http://www.midi-inc.com/) (MIDI, 2005) and the Microbial Identification software package (MIDI, 2005). Menaquinones, cell wall diamino acids, and whole-cell sugars were analyzed as previously described (Xu et al., 2007; Ruan and Huang, 2011). Polar lipids of strain HUAS TT1^T^ and *S. longispora* CGMCC 4.1357^T^ were extracted and analyzed as described by Ruan and Huang (2011). All strains were cultivated under the same conditions and detected using the same method.

### 2.4 Genome sequencing, annotation, and phylogeny

Genome sequencing of strain HUAS TT1^T^ was performed by Wuhan Benagen Technology Co., Ltd. (Hubei, PR China). Detailed methods have been described by Chen et al. (2022). Gene prediction, functional annotation, and secondary metabolism biosynthetic gene cluster analysis of the genomes of strains HUAS TT1^T^, *S. hoggarensis* DSM 45457^T^, *S. saharensis* DSM 45456^T^, *S. yanglingensis* CGMCC 4.5627^T,^ and *S. longispora* CGMCC 4.1357 ^T^ were performed by Rapid Annotation using Subsystem Technology server version 2.0 (https://rast.nmpdr.org, accessed on 20 July 2024) (Aziz et al., 2008) and antiSMASH version 6.0.1^3^ (Makarova et al., 2019). The complete 16S rRNA gene sequence (1,519 bp) of strain HUAS TT1^T^ was obtained from the whole genome and then blasted in the EzBioCloud server (www.ezbiocloud.net/identify, accessed on 20 July 2024). Based on the search results, the 16S rRNA gene sequences of strain HUAS TT1^T^ and its related strains were selected to construct maximum-likelihood (ML) (Felsenstein, 1981), maximum-parsimony (MP) (Kluge and Farris, 1969), and neighbor-joining (NJ) (Saitou and Nei, 1987) phylogenetic trees using the MEGA 11 software package (Tamura et al., 2021) with 1,000 bootstrap repeats. The NJ and ML trees were constructed using the Kimura 2-parameter model (Kimura, 1980). The MP tree was constructed using the Subtree-Pruning-Regrafting (SPR) algorithm (Nei and Kumar, 2000). The Type (Strain) Genome Server (https://tygs.dsmz.de/, accessed on 20 July 2024) was used to reconstruct the phylogenomic tree (Meier-Kolthoff et al., 2019). Meanwhile, for genomic analyses, genome sequences of each gene cluster were aligned with MUSCLE v5 (Edgar, 2022), the default multiple sequence alignment tool integrated in EasyCGTree v4.2 (Zhang et al., 2023), and low-confidence regions in the alignments were filtered using trimAl v1.2 (Capella-Gutiérrez et al., 2009) under the “strict” criterion (EasyCGTree v4.2’s default trimming strategy). A maximum likelihood (ML) phylogenomic tree was inferred with FastTree 2.1 (Price et al., 2009) based on concatenated sequences of the bac120 core-gene set (post-trimming and alignment). The final tree was visualized using tvBOT (Xie et al., 2023) in accordance with the tool’s official operational guidelines. The genomes of the type strains used for reconstructing a phylogenomic tree were obtained from the EzBioCloud server or NCBI, and their genome quality must meet the criteria of >95% completeness and <5% contamination to obtain more reliable results (Parks et al., 2015). Quality analysis and GenBank assembly of the genomes are shown in Supplementary Table S1. The average nucleotide identity (ANI) based on BLAST+(ANIb, BLAST+2.11.0) and MUMmer (ANIm) and digital DNA–DNA hybridization (dDDH) with formula d4 values between the genomes of strain HUAS TT1^T^ and its related type strains was computed using the JSpeciesWS online service (https://jspecies.ribohost.com/jspeciesws/#analyse, © 2014–2025. Ribocon GmbH-Version: 5.0.3, accessed on 30 November 2025, Richter et al., 2016) and the Genome-to-Genome Distance Calculator 3.0 with local alignment tool (BLAST+, recommended) (http://ggdc.dsmz.de/distcalc2.php, accessed from 30 November 2025 to 3 December 2025, Meier-Kolthoff et al., 2013), respectively.

## 3 Results and discussion

### 3.1 Phenotypic characteristics

**Figure 1:**
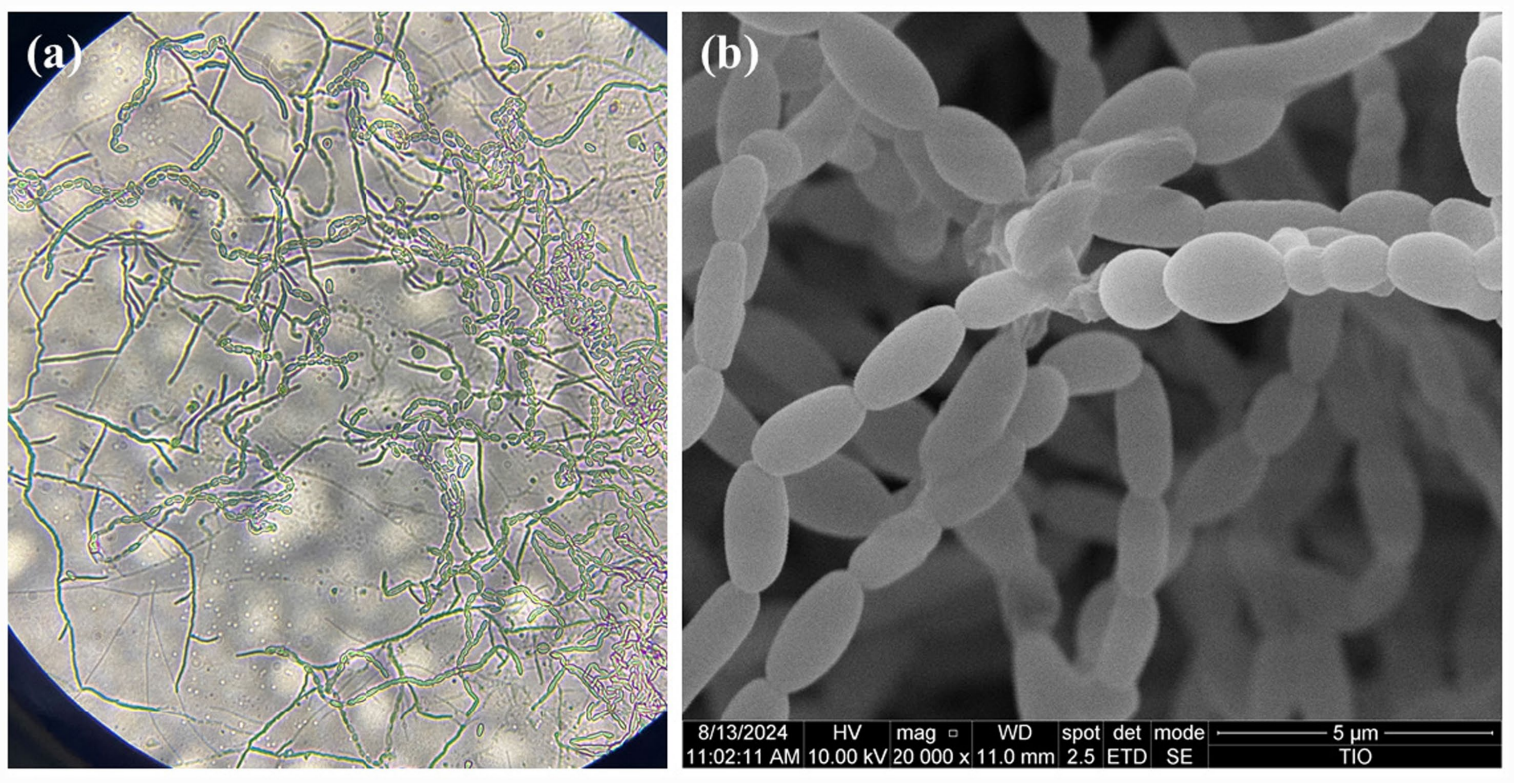
Light microscope (100 × 40) **(a)** and scanning electron microscope images **(b)** of strain HUAS TT1^T^ grown on R2A medium after incubation for 14 days at 28 °C.

Strain HUAS TT1^T^ formed white aerial mycelia and light vinaceous-cinnamon substrate mycelia with cinnamon-buff diffusible pigments on R2A medium. Mature aerial mycelia produced slightly flexuous spore chains consisting of rod-shaped spores with smooth surfaces (Figure 1). Strain HUAS TT1^T^ grew well in all tested media and produced diffusible pigments on all tested media (Supplementary Table S2). Strain HUAS TT1^T^ grew at 20–30 °C (optimum, 28 °C), pH 6.0–8.0 (optimum, pH 7.0), and in the presence of 3.0% (w/v) NaCl (optimum, 1.0%). Strain HUAS TT1^T^ was positive for gelatin liquefaction but negative for hydrolysis of starch, nitrate reduction, and decomposition of Tweens (20, 40, 60, 80) (Table 1).

**Table 1:** Characteristics of strains HUAS TT1^T^, *S. hoggarensis* DSM 45457^T^, *S. saharensis* DSM 45456^T^, *S. yanglingensis* CGMCC 4.5627^T^, and *S. longispora* CGMCC 4.1357^T^.

Characteristic	1	2	3	4	5
BiologGEN III test					
Acetic acid, acetoacetic acid, aztreonam, bromo-succinic acid, d-arabitol, d-cellobiose, dextrin, d-fructose, d-fructose-6-PO_4_, d-galactose, d-galacturonic acid, d-gluconic acid, d-glucuronic acid, d-malic acid, d-maltose, d-mannitol, d-mannose, d-melibiose, d-raffinose, d-saccharic acid, d-salicin, d-serine, d-sorbitol, d-trehalose, d-turanose, gelatin, gentiobiose, glucuronamide, glycerol, glycyl-l-proline, inosine, l-alanine, l-arginine, l-aspartic acid, l-fucose, l-galactonic acid lactone, l-glutamic acid, l-lactic acid, l-pyroglutamic acid, l-rhamnose, *myo*-inositol, *N*-acetyl-d-galactosamine, *N*-acetyl-d-glucosamine, *N*-acetylneuraminic acid, nalidixic acid, potassium tellurite, propionic acid, quinic acid, rifamycin SV, sodium bromate, stachyose, *α*-d-glucose, *α*-d-lactose, *α*-hydroxy-butyric acid, *α*-Keto-glutaric acid, *β*-hydroxy-d, l-butyric acid, *β*-methyl-d-glucoside, *γ*-amino-butryric acid, 1% sodium lactate, 3-methyl glucose	+	+	−	+	+
d-Aspartic acid, d-fucose, d-lactic acid methyl ester, formic acid, *N*-acetyl-*β*-dmannosamine, sucrose, *α*-keto-butyric acid,	+	−	+	+	+
Methyl pyruvate, sodium butyrate	W	−	−	W	W
Citric acid, p-hydroxy-phenylacetic acid	+			W	W
Mucic acid	−			W	W
Minocycline	−			+	W
Fusidic acid, Niaproof 4, tetrazolium blue	−			−	−
Guanidine HCl, lincomycin, l-phenylalanine, pectin, vancomycin, *α*-keto-butyric acid,	−	+	+	+	+
l-Ornithine, tetrazolium violet, troleandomycin	−	+	−	+	+
d-Glucose-6-PO_4_, lithium chloride, l-malic acid	+	+	−	−	+
l-Histidine, l-serine, *α*-keto-glutaric acid	+	−	−	−	+
API ZYM test					
Acid phosphatase, cystine arylamidase, esterase (C4), trypsin, leucine arylamidase, lipase (C14), *N*-acetyl-*β*-glucosaminidase,	+	−	−	−	−
Alkaline phosphatase, esterase lipase (C8),	+	−	−	−	−
Naphtol-AS-BI-phosphohydrolase, valine arylamidase, *α*-chymotrypsin, *α*-fucosidase, *α*-mannosidase,	+	+	−	−	−
*α*-Galactosidase, *α*-glucosidase,*β*-glucuronidase	−	+	+	+	+
*β*-Galactosidase, *β*-glucosidase,	−	+	+	+	+
Hydrolysis of starch, nitrate reduction	−	+	+	+	+
Gelatin liquefaction	+	−	−	−	−
Decomposition of Tweens 20, 40, 60, 80	−	+	+	+	+
Growth at/in:					
Temperature (°C)	20–30 (28)	15–45 (30)	20–35 (30)	15–35 (28)	15–35 (28)
NaCl tolerance (%, w/v)	0–3.0 (1.0)	0–5.0 (1.0)	0–2.0 (1.0)	0–2.0 (1.0)	0–4.0 (1.0)
pH	6.0–8.0 (7.0)	7.0–9.0 (7.0)	5.0–9.0 (7.0)	5.0–9.0 (7.0)	5.0–9.0 (7.0)
Menaquinones	MK-9(H_4_) (50.3%)	MK-9(H_4_) (62%)	MK-9(H_4_) (70%)	MK-9(H_4_) (80.5%)	MK-9(H_4_) (86.1%)
		MK-7(H_4_) (23%)	MK-7(H_4_) (20%)		
	MK-9(H_8_) (35.5%)				
		MK-10(H_4_) (8.5%)			
					MK-10(H_4_) (12.9%)
				MK-10(H_4_) (17.8%)	
	MK-9(H_2_) (12.3%)				
		MK-10(H_0_) (4.7%)			
Cell-wall diamino acid	*mes*-DAP	*mes*-DAP	*mes*-DAP	*mes*-DAP	*mes*-DAP
Whole-cell sugars	Galactose, ribose	Galactose, mannose, rhamnose, ribose	Galactose, mannose, rhamnose, ribose	Galactose, mannose	Galactose, mannose
Polar lipids	PE, PI, PIM,	PE, PI, PME,	PE, PME^b^	PE, DPG^c^	PE, DPG
	DPG, OH-PE	PIM, DPG^a^			

1, HUAS TT1^T^; 2, *S. hoggarensis* DSM 45457^T^; 3, *S. saharensis* DSM 45456^T^; 4, *S. yanglingensis* CGMCC 4.5627^T^; 5, *S. longispora* CGMCC 4.1357^T^. W, positive weak; +, positive; −, negative; mes-DAP, meso-diaminopimelic acid. PE, Phosphatidylethanolamine; PI, Phosphatidylinositol; PME, Phosphatidylmethylethanolamine; PIM, Phosphatidyl inositol mannosides; DPG, Diphosphatidylglycerol; OH-PE, hydroxy phosphatidylethanolamine. All data are obtained from this study, except for those cited. All data are from this study except cited. a, Data from Dalila et al. (2013a). b, Data from Dalila et al. (2013b). c, Data from Yan et al. (2012).

### 3.2 Chemotaxonomic characterization

The polar lipids in strain HUAS TT1^T^ included diphosphatidylglycerol (DPG), hydroxy phosphatidylethanolamine (OH-PE), phosphatidylethanolamine (PE), phosphatidylinositol (PI), and phosphatidylinositol mannosides (PIM) (Supplementary Figure S1). The major fatty acids (>10.0%) of strain HUAS TT1^T^ were *iso*-C_16:0_ (14.7%), Summed Feature 5 (C_18:2_ ω6, 9c/C_18:0_ ante) (14.6%), C_18:1_ ω9c (14.0%), C_16:0_ (11.3%), and *iso*-C_15:0_ (11.1%) (Supplementary Figure S2, Supplementary Tables S3, S4). The cell wall of strain HUAS TT1^T^ contained *meso*-diaminopimelic acid, and whole-cell hydrolysates contained galactose and ribose. The menaquinones in strain HUAS TT1^T^ were MK-9(H_4_) (50.3%), MK-9(H_8_) (35.5%), and MK-9(H_2_) (12.3%) (Table 1).

### 3.3 Genomic characterization

The DNA G+C content of the genome sequence, consisting of 8,515,408 bp, was 72.7%. A total of 7,761 genes (7,581 coding genes, 87 RNA genes, and 93 pseudogenes) and 7,674 CDSs (7,581 CDSs with protein and 93 CDSs without protein) were predicted (Supplementary Table S5). The annotated genome of strain HUAS TT1^T^ had 309 subsystems divided into 20 categories, with a subsystem coverage of 17%. They represented up to seven subsystem features, identified as ‘Amino Acids and Derivatives’ (392 CDSs), ‘Carbohydrates’ (319 CDSs), ‘Protein Metabolism’ (203 CDSs), ‘Cofactors, Vitamins, Prosthetic Groups, Pigments’ (175 CDSs), ‘Fatty Acids, Lipids, and Isoprenoids’ (164 CDSs), ‘Nucleosides and Nucleotides’ (104 CDSs), and ‘DNA Metabolism’ (103 CDSs) (Supplementary Table S6).

The results of the antiSMASH analysis showed that the genome of strain HUAS TT1^T^ included five main biosynthetic gene clusters: T1PKS (Type I PKS polyketide synthase), NRPS (non-ribosomal peptide synthetase), terpene, PKS-like (other types of PKS), and NRP-metallophore (Supplementary Table S7, Supplementary Figure S3).

### 3.4 Phylogeny and DNA–DNA correlation analysis

**Figure 2:**
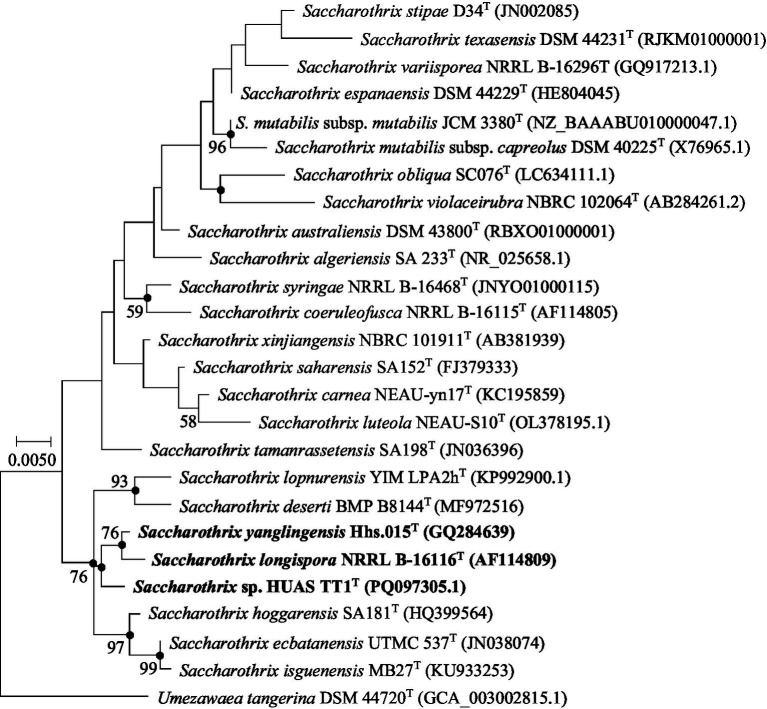
Maximum-likelihood phylogenetic tree based on 16S rRNA gene sequences showing the relationship between selected species of *Saccharothrix*. *Umezawaea tangerina* DSM 44720^T^ was used as an outgroup. Bootstrap percentages of over 50% derived from 1,000 replications are shown at the nodes. Dots indicate branches also recovered in the neighbor-joining and maximum-parsimony trees. Scale bar: 0.0050 substitutions per site.

**Figure 3:**
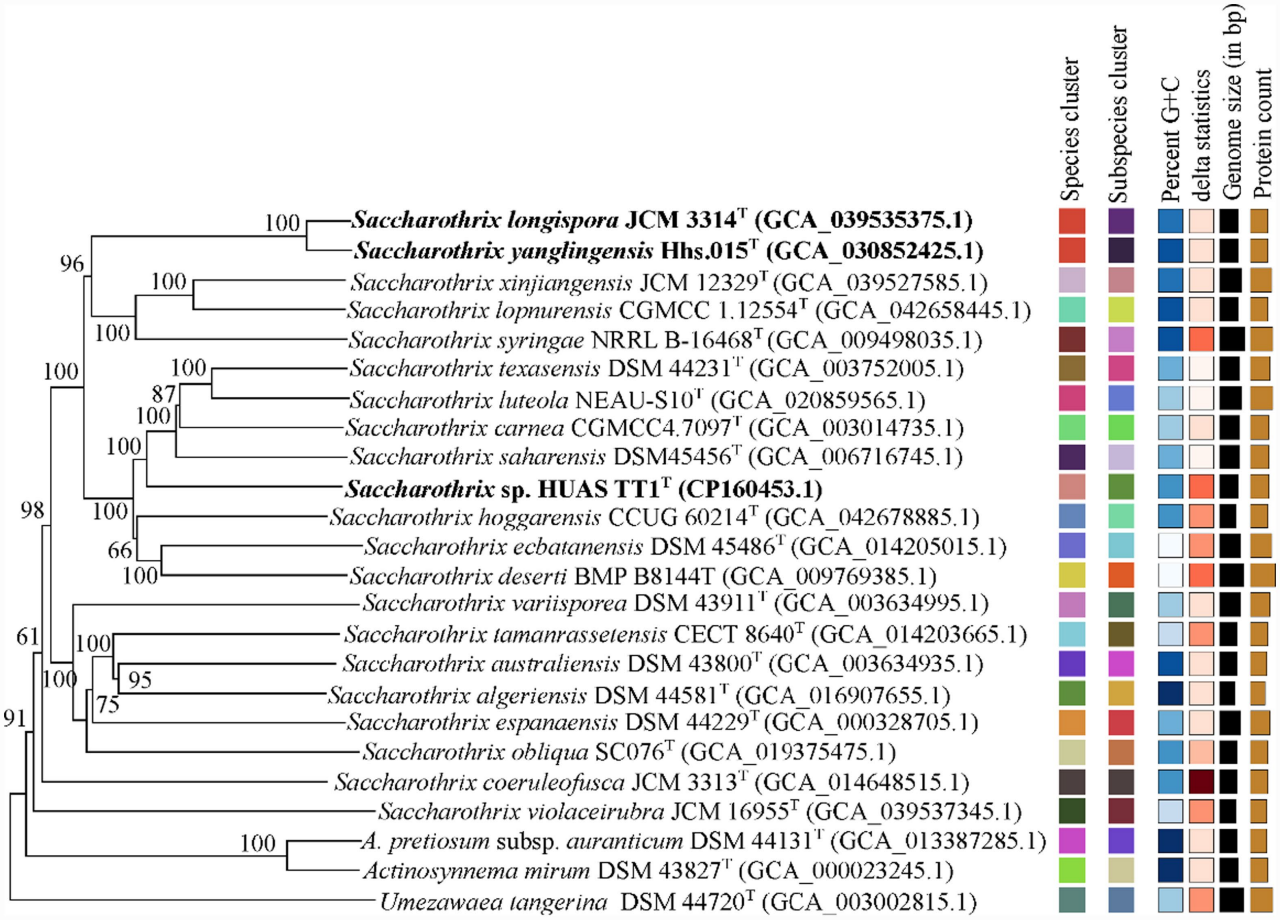
Phylogenetic tree based on whole-genome sequences of strain HUAS TT1^T^ and related reference strains. The tree inferred with FastME 2.1.6.1 (Farris, 1972) from GBDP distances calculated from genome sequences. The branch lengths are scaled in terms of the GBDP distance formula d5. The numbers above the branches are GBDP pseudo-bootstrap support values of >60% from 100 replications, with an average branch support of 96.0%. The tree was rooted at the midpoint (Vincent et al., 2015).

Full-length 16S rRNA gene sequence (1,519 bp) analysis of strain HUAS TT1^T^ revealed that it belonged to the genus *Saccharothrix,* with the highest sequence similarities to *S. yanglingensis* Hhs.015^T^ (99.30%), *S. carnea* NEAU-yn17^T^ (98.89%), *S. hoggarensis* SA181^T^ (98.89%), *S. saharensis* SA152^T^ (98.82%), *S. longispora* NRRL B-16116^T^ (98.75%), and *S. ecbatanensis* UTMC 537^T^ (98.61%). Phylogenetic trees based on 16S rRNA gene sequences showed that strain HUAS TT1^T^ was related to *S. yanglingensis* Hhs.015^T^ and *S. longispora* NRRL B-16116^T^ (Figure 2; Supplementary Figures S4, S5). However, phylogenetic trees based on whole-genome and 120 core gene sequences demonstrated that the evolutionary neighbors of strain HUAS TT1^T^ were *S. hoggarensis* CCUG 60214^T^ and *S. saharensis* DSM45456^T^ cells (Figure 3, Supplementary Figure S6), suggesting that phylogenetic analyses based on both genomes and 120 core genes could provide better resolution than phylogenetic analyses based on 16S rRNA gene sequences. This result is consistent with previous findings (Rodriguez et al., 2018). Based on this fact, the DNA–DNA reassociation between strain HUAS TT1^T^ and *S. hoggarensis* CCUG 60214^T^/*S. Saharensis* DSM45456^T^ was also evaluated. The results indicated that the ANIb/m and dDDH values of strain HUAS TT1^T^ and these two strains were 85.82%/88.80 and 32.90%, 86.42%/89.43, and 34.40% (Table 2, Supplementary Table S8), respectively, much less than the 95–96 and 70% cut-off points recommended for delineating species (Richter and Rosselló-Móra, 2009; Wayne et al., 1987), suggesting that strain HUAS TT1^T^ and they belonged to three different species. This was also confirmed by a comprehensive comparison of phenotypic and chemotaxonomic characteristics between strain HUAS TT1^T^ and the two strains *S. hoggarensis* DSM 45457^T^ and *S. saharensis* DSM 45456^T^ (Table 1, Supplementary Tables S2–S5, Supplementary Figures S2, S3). In addition, as proposed by Stackebrandt and Ebers (2006), if two strains share ≥98.7% 16S rRNA gene sequence similarity, their ANI and dDDH values should be calculated. In view of the type strains *S. yanglingensis* Hhs.015^T^, *S. carnea* NEAU-yn17^T^, and *S. longispora* NRRL B-16116^T^, which exhibited ≥98.7% 16S rRNA gene sequence similarity to strain HUAS TT1^T^, ANI and dDDH should be calculated. However, the ANIb/m and dDDH values between the genome sequences of strain HUAS TT1^T^ were well below the 95–96 and 70% cut-off points recommended for delineating species (Table 2, Supplementary Table S8).

**Table 2:** Comparative genotypic analysis between strain HUAS TT1^T^ and the closest related type strains, *S. yanglingensis* Hhs.015^T^, *S. carnea* CGMCC4.7097^T^, *S. hoggarensis* CCUG 60214^T^, *S. saharensis* DSM45456^T^, and *S. longispora* JCM 3314^T^.

Closest related species	Strain HUAS TT1^T^			
	16S rRNA gene sequence similarity	ANIb	ANIm	dDDH
*Saccharothrix yanglingensis* Hhs.015^T^	99.30%	83.31%	87.39%	28.70%
*Saccharothrix carnea* CGMCC4.7097^T^	98.89%	86.26%	89.46%	34.70%
*Saccharothrix hoggarensis* CCUG 60214^T^	98.89%	85.82%	88.80%	32.90%
*Saccharothrix saharensis* DSM45456^T^	98.82%	86.42%	89.43%	34.40%
*Saccharothrix longispora* JCM 3314^T^	98.75%	83.10%	87.25%	28.50%

Based on the above data, strain HUAS TT1^T^ represents a novel species of the genus *Saccharothrix*, for which the name *Saccharothrix camelliae* sp. nov. is proposed.

In addition, *Saccharothrix longispora*, as a novel species, was first described as *Actinomadura longispora* in 1974 (Preobrazhenskaya and Sveshnikova, 1974) and approved in 1980 (Skerman et al., 1980). *Actinomadura longispora* was transferred to the genus *Nocardiopsis* in 1982 (Preobrazhenskaya et al., 1982) and was validated in 1985 (Anonymous, 1985). In 1989, *Nocardiopsis longispora* was transferred to the genus *Saccharothrix* based on chemotaxonomy and numerical taxonomy (Grund and Kroppenstedt, 1989) and was validated in 1990 (Anonymous, 1990). Another new species, *Saccharothrix yanglingensis,* was first reported in 2012 (Yan et al., 2012) and validated in 2025 (Oren and Göker, 2025). However, in the course of determining the taxonomic status of strain HUAS TT1^T^, it was found that the ANIb/m and dDDH values between the genome sequences of *S. yanglingensis* Hhs.015^T^ and *S. longispora* JCM 3314^T^ were 96.43%/97.35 and 75.10% (Supplementary Table S8), respectively, well above the 95–96 and 70% cut-off points recommended for delineating species (Richter and Rosselló-Móra, 2009; Wayne et al., 1987). Moreover, this result was further confirmed by a comparison of their phenotypic characteristics. Most features of *S. yanglingensis* CGMCC 4.5627^T^ and *S. longispora* CGMCC 4.1357^T^ were similar (Table 1, Supplementary Tables S2–S5, Supplementary Figures S2, S3). For example, in Biolog GEN III tests, both *S. yanglingensis* CGMCC 4.5627^T^ and *S. longispora* CGMCC 4.1357^T^ showed positive for a wide range of substrates including acetic acid, acetoacetic acid, aztreonam, bromo-succinic acid, d-arabitol, d-aspartic acid, d-cellobiose, dextrin, d-fructose, d-fructose-6-PO_4_, d-fucose, d-galactose and so on, and negative for fusidic acid, Niaproof 4, tetrazolium blue; in API ZYM test, both of them were positive for *α*-galactosidase, *α*-glucosidase, *β*-galactosidase, *β*-glucosidase, *β*-glucuronidase, and negative for acid phosphatase, alkaline phosphatase, cystine arylamidase, esterase (C4), esterase lipase (C8), trypsin, leucine arylamidase, lipase (C14), *N*-acetyl-*β*-glucosaminidase, naphtol-AS-BI-phosphohydrolase, valine arylamidase, *α*-chymotrypsin, *α*-fucosidase and *α*-mannosidase. They also share similar growth conditions, with an optimal temperature of 28 °C within the range of 15–35 °C, NaCl tolerance up to 2.0 and 4.0%, respectively, and a pH range of 5.0–9.0 with an optimal pH of 7.0. Both strains have *mes*-DAP as the cell wall diamino acid and galactose and mannose as the whole-cell sugars. Additionally, menaquinones include MK-9 (H_4_) as a major component. *S. yanglingensis* CGMCC 4.5627^T^ and *S. longispora* CGMCC 4.1357^T^ also exhibited similarities in fatty acid composition, with both having notable levels of *iso*-C_16:0_, C_17:1_ ω6c, and *iso*-C_15:0_. They also shared the presence of *iso*-C_15:1_ G and C_18:1_ ω9c. Therefore, based on these results and Rule 42 of the Bacteriological Code (Garrity et al., 2019), we propose that *Saccharothrix yanglingensis* Yan et al. 2012 is a later heterotypic synonym of *Saccharothrix longispora* (Preobrazhenskaya and Sveshnikova, 1974; Grund and Kroppenstedt, 1990).

## 4 Descriptions

### 4.1 Description *Saccharothrix camelliae* sp. nov

*Saccharothrix camelliae* (ca.mel’li.ae. N. L. gen. n. *camelliae* of *Camellia*, referring to the isolation of the type strain from *Camellia oleifera* Abel).

Forms white aerial mycelium and light vinaceous-cinnamon substrate mycelium with cinnamon-buff diffusible pigment on R2A medium. Aerobic, Gram-stain-positive actinomycete that forms slightly flexuous spore chains consisting of rod-shaped spores with smooth surfaces. It grows well on all tested media (Gause′s synthetic No.1 medium, R2A medium, and ISP 2–7) and produces diffusible pigments on all tested media. Growth occurs at 20–30 °C (optimum, 28 °C), pH 6.0–8.0 (optimum, pH 7.0), and in the presence of 3.0% (w/v) NaCl (optimum, 1.0%). It is positive for gelatin liquefaction but negative for hydrolysis of starch, nitrate reduction, and decomposition of Tweens (20, 40, 60, 80). In Biolog GEN III tests, positive for growth on acetic acid, acetoacetic acid, aztreonam, bromo-succinic acid, citric acid d-arabitol, d-aspartic acid, d-cellobiose, dextrin, d-fructose, d-fructose-6-PO_4_, d-fucose, d-galactose, d-galacturonic acid, d-glucose-6-PO_4_, d-gluconic acid, d-glucuronic acid, d-lactic acid methyl ester, d-malic acid, d-maltose, d-mannitol, d-mannose, d-melibiose, d-raffinose, d-saccharic acid, d-salicin, d-serine, d-sorbitol, d-trehalose, d-turanose, formic acid, gelatin, gentiobiose, glucuronamide, glycerol, glycyl-l-proline, inosine, l-alanine, l-arginine, l-aspartic acid, l-fucose, l-galactonic acid lactone, l-glutamic acid, l-histidine, l-lactic acid, lithium chloride, l-malic acid, l-serine, l-pyroglutamic acid, l-rhamnose, myo-inositol, *N*-acetyl-d-galactosamine, *N*-acetyl-d-glucosamine, *N*-acetylneuraminic acid, *N*-acetyl-*β*-dmannosamine, nalidixic acid, p-hydroxy-phenylacetic acid, potassium tellurite, propionic acid, quinic acid, rifamycinsv, sodium bromate, stachyose, sucrose, *α*-d-glucose, *α*-d-lactose, *α*-hydroxy-butyric acid, *α*-keto-butyric acid, *α*-Keto-glutaric acid, *β*-hydroxy-d, l-butyric acid, *β*-methyl-d-glucoside, *γ*-amino-butryric acid, 1% sodium lactate, and 3-methyl glucose; but negative for growth on fusidic acid, guanidine HCl, lincomycin, l-ornithine, l-phenylalanine, minocycline, mucic acid, Niaproof 4, pectin, tetrazolium blue, tetrazolium violet, troleandomycin, vancomycin, and *α*-keto-butyric acid. API ZYM test, positive for acid phosphatase, alkaline phosphatase, cystine arylamidase, esterase (C4), esterase lipase (C8), trypsin, leucine arylamidase, lipase (C14), *N*-acetyl-*β*-glucosaminidase, naphtol-AS-BI-phosphohydrolase, valine arylamidase, *α*-chymotrypsin, *α*-fucosidase, and *α*-mannosidase; but negative for *α*-galactosidase, *α*-glucosidase, *β*-galactosidase, *β*-glucosidase, and *β*-glucuronidase. The polar lipids are diphosphatidylglycerol (DPG), hydroxy phosphatidylethanolamine (OH-PE), phosphatidylethanolamine (PE), phosphatidylinositol (PI), and phosphatidylinositol mannosides (PIM). The major fatty acid composition (>10.0%) is *iso*-C_16:0_, Summed Feature 5 (C_18:2_ ω6, 9c/C_18:0_ ante), C_18:1_ ω9c, C_16:0,_ and *iso*-C_15:0_. The cell wall contains *meso*-diaminopimelic acid, and the whole-cell hydrolysates are galactose and ribose. The menaquinones are MK-9(H_4_), MK-9(H_8_), and MK-9(H_2_).

The type strain was HUAS TT1^T^ (=MCCC 1K09364^T^ = JCM 37292^T^), which was isolated from the rhizosphere soil of *Camellia oleifera* Abel collected from Changde City, Hunan Province, PR China. The DNA G+C content of the genome sequence, consisting of 8,515,408 bp, is 72.7 mol%. The NCBI accession numbers for the 16S rRNA gene sequence and genome sequence of strain HUAS TT1^T^ are PQ097305.1 and CP160453.1, respectively. Raw reads of strain HUAS TT1^T^ were deposited in the Sequence Read Archive (SRA) under NCBI accession numbers. PRJNA1132529.

### 4.2 Emended description of *Saccharothrix longispora*

Heterotypic synonym: *Saccharothrix yanglingensis* (Yan et al. 2012).

The description is as before (Preobrazhenskaya and Sveshnikova, 1974; Grund and Kroppenstedt, 1990), with the following additions. Growth occurs at 15–35 °C (optimum, 28 °C), pH 5.0–9.0 (optimum, pH 7.0), and in the presence of 4.0% (w/v) NaCl (optimum, 1.0%). Positive for hydrolysis of starch, nitrate reduction, and decomposition of Tweens (20, 40, 60, and 80), but negative for gelatin liquefaction. In Biolog GEN III tests, positive for acetic acid, acetoacetic acid, aztreonam, bromo-succinic acid, d-arabitol, d-aspartic acid, d-cellobiose, dextrin, d-fructose, d-fructose-6-PO_4_, d-fucose, d-galactose, d-galacturonic acid, d-gluconic acid, d-glucose-6-PO_4_, d-glucuronic acid, d-lactic acid methyl ester, d-malic acid, d-maltose, d-mannitol, d-mannose, d-melibiose, d-raffinose, d-saccharic acid, d-salicin, d-serine, d-sorbitol, d-trehalose, d-turanose, guanidine HCl, formic acid, gelatin, gentiobiose, glucuronamide, glycerol, glycyl-l-proline, inosine, l-alanine, l-arginine, l-aspartic acid, l-fucose, l-galactonic acid lactone, l-glutamic acid, l-histidine, l-lactic acid, lincomycin, lithium chloride, l-malic acid, l-ornithine, l-phenylalanine, l-pyroglutamic acid, l-rhamnose, l-serine, *myo*-inositol, *N*-acetyl-d-galactosamine, *N*-acetyl-d-glucosamine, *N*-acetylneuraminic acid, *N*-acetyl-*β*-dmannosamine, nalidixic acid, pectin, potassium tellurite, propionic acid, quinic acid, rifamycinsv, sodium bromate, stachyose, sucrose, vancomycin, tetrazolium violet, troleandomycin, *α*-d-glucose, *α*-d-lactose, *α*-hydroxy-butyric acid, *α*-keto-butyric acid, *α*-Keto-glutaric acid, *β*-hydroxy-d, l-butyric acid, *β*-methyl-d-glucoside, *γ*-amino-butryric acid, 1% sodium lactate, and 3-methyl glucose; but negative for fusidic acid, Niaproof 4, and tetrazolium blue. API ZYM test, positive for *α*-Galactosidase, *α*-glucosidase, *β*-galactosidase, *β*-glucosidase, and *β*-glucuronidase, but negative for acid phosphatase, alkaline phosphatase, cystine arylamidase, esterase (C4), esterase lipase (C8), trypsin, leucine arylamidase, lipase (C14), *N*-acetyl-*β*-glucosaminidase, naphtol-AS-BI-phosphohydrolase, valine arylamidase, *α*-chymotrypsin, *α*-fucosidase, and *α*-mannosidase. The major fatty acids (>10.0%) were *iso*-C_16:0_, C_17:1_ ω6c, and Summed Feature 3 (C_16:1_ ω7c/C_16:1_ ω9c). The cell wall contains *meso*-diaminopimelic acid, and the whole-cell hydrolysates are galactose and mannose. The menaquinones used were MK-9(H_4_) and MK-10(H_4_). Polar lipids include phosphatidylethanolamine and diphosphatidylglycerol.

The DNA G+C content of the genome sequence, consisting of 8,393,791 bp, is 73.0 mol%. The GenBank accession number for the whole-genome sequence is GCA_039535375.1. The type strain is CGMCC 4.1357^T^ (=JCM 3314^T^ =ATCC 35109^T^ =BCRC 13395^T^ =DSM 43749^T^ =HUT 6594^T^ =IFO 14522^T^ =IMET 9603^T^ =IMSNU 21359^T^ =INA 10222^T^ =KCTC 9394^T^ =NBRC 14522^T^ = NRRL B-16116^T^ =VKM Ac-907^T^).

## Data Availability

The NCBI accession numbers for the 16S rRNA gene sequence and genome sequence of strain HUAS TT1T are PQ097305.1 and CP160453.1, respectively. The raw reads of strain HUAS TT1T were deposited in the Sequence Read Archive (SRA) under the NCBI accession numbers PRJNA1132529.

## Footnotes

1
https://lpsn.dsmz.de/genus/saccharothrix

2
www.npatlas.org

3
https://antismash.secondarymetabolites.org
