# Supplementary material for: Saccharothrix camelliae sp. nov., isolated from rhizosphere soil of Camellia oleifera Abel and proposal of Saccharothrixyanglingensis as a later heterotypic synonym of Saccharothrix longispora
Source: Front Microbiol. 2026 Feb 27;17:1716500. doi: 10.3389/fmicb.2026.1716500 (PMC12983401; doi:10.3389/fmicb.2026.1716500)
Supplement: Supplementary file 1 [file Data_Sheet_1.PDF]

JAPAN COLLECTION OF MICROORGANISMS

Microbe Division, RIKEN BioResource Research Center
3-1-1 Koyadai, Tsukuba, Ibaraki 305-0074, Japan

Phone : +81 29 836 9556
Fax : +81 29 836 9561
E-mail : inquiry.jcm@riken.jp

No. 240425
12 Nov 2024

# CERTIFICATE OF DEPOSITION AND AVAILABILITY OF A MICROORGANISM

This is to certify that the following microorganism has been deposited into the Japan Collection of Microorganisms (JCM) and will be available to the public without restriction after publication by the author(s) of the scientific paper that describes this taxon:

JCM 37292 *Saccharothrix* sp.
<- P. Mo; Hunan Univ. of Arts & Sci., China; HUAS TT1

Moriya Ohkuma, Ph.D.
Director
Microbe Division / Japan Collection of Microorganisms
The RIKEN BioResource Research Center

***NOTICE:*** *This document is valid only for the proposal of a new scientific name with the above-mentioned strain in the International Journal of Systematic and Evolutionary Microbiology (IJSEM). The scientific name is based on the registered one as of the issue date, and any later update of the name does not invalidate this document. JCM does not reissue this certificate in principle, unless the MTA (for deposition) in connection with the strain is renewed.*

国立研究開発法人 理化学研究所 バイオリソース研究センター 微生物材料開発室
〒305-0074 茨城県つくば市高野台3丁目1番地1
