# Supplementary material for: Saccharothrix camelliae sp. nov., isolated from rhizosphere soil of Camellia oleifera Abel and proposal of Saccharothrixyanglingensis as a later heterotypic synonym of Saccharothrix longispora
Source: Front Microbiol. 2026 Feb 27;17:1716500. doi: 10.3389/fmicb.2026.1716500 (PMC12983401; doi:10.3389/fmicb.2026.1716500)
Supplement: Supplementary file 2 [file Data_Sheet_2.PDF]

国家菌种资源库 National Microbial Resource Center

中国海洋微生物菌种保藏管理中心
Marine Culture Collection of China

CERTIFICATE OF DEPOSIT

IN MARINE CULTURE COLLECTION OF CHINA

Marine Culture Collection of China
Third Institute of Oceanography, Ministry of Natural Resources
No. 178 Daxue Road, 361005 Xiamen, Fujian Province
P. R. China.
Phone/Fax: +86-592-2195177
Email: mccc5177@163.com
Web site: http://www.mccc.org.cn

MCCC 1K09364

***Saccharothrix* sp. (strain HUAS TT1) was received for deposit**
in Marine Culture Collection of China from

Ping Mo

Hunan University of Arts and Science
No. 3150, Dongting Avenue, 415000 Changde, Hunan Province
P. R. China

on June 4, 2024

and was, after confirming the viability and purity,
allocated the accession number MCCC 1K09364.

The strain is available to any bona fide scientific community or individual,
operating in a professional environment
suitable for handling living material of the biohazard group involved.

Xiamen, July 18, 2024

Zongze Shao

Dr. Zongze Shao
Public Collection Curator
Marine Culture Collection of China
