# Supplementary material for: Saccharothrix camelliae sp. nov., isolated from rhizosphere soil of Camellia oleifera Abel and proposal of Saccharothrixyanglingensis as a later heterotypic synonym of Saccharothrix longispora
Source: Front Microbiol. 2026 Feb 27;17:1716500. doi: 10.3389/fmicb.2026.1716500 (PMC12983401; doi:10.3389/fmicb.2026.1716500)
Supplement: Supplementary file 3 [file Data_Sheet_3.PDF]

Saccharothrix camelliae sp. nov., isolated from rhizosphere soil of *Camellia oleifera* Abel and proposal of *Saccharothrix yanglingensis* Yan et al. 2012 as a later heterotypic synonym of *Saccharothrix longispora* (Preobrazhenskaya and Sveshnikova 1974) Grund and Kroppenstedt 1990

Ting Tang[1†], Hang Jia[1, 2†], Yuhuan Cao[1, 2], Jia Deng[1],

Mingjun Ke[1], Ping Mo[1, 2, 3]*, Kaiqin Li[4]*, Jian Gao[4]

Author affiliations:

[1]Key Laboratory of Agricultural Products Processing and Food Safety in Hunan Higher Education, Hunan Provincial Engineering Research Center for Fresh Wet Rice Noodles, Science and Technology Innovation Team for Efficient Agricultural Production and Deep Processing at General University in Hunan Province, Changde Key Innovation Team for wetland biology and Environmental Ecology, College of Life and Environmental Sciences, Hunan University of Arts and Science, Changde 415000, Hunan Province, People's Republic of China.

[2]College of Furong, Hunan University of Arts and Science, Changde 415000, Hunan Province, PR China.

[3]College of Synthetic Biology Industry, Hunan University of Arts and Science, Changde 415000, China.

[4]Hunan University of Science and Technology, Xiangtan 411201, China.

†These authors contributed equally to this work.

*Correspondence:

Ping Mo, moping2015@126.com; Kaiqin Li, 782649249@qq.com

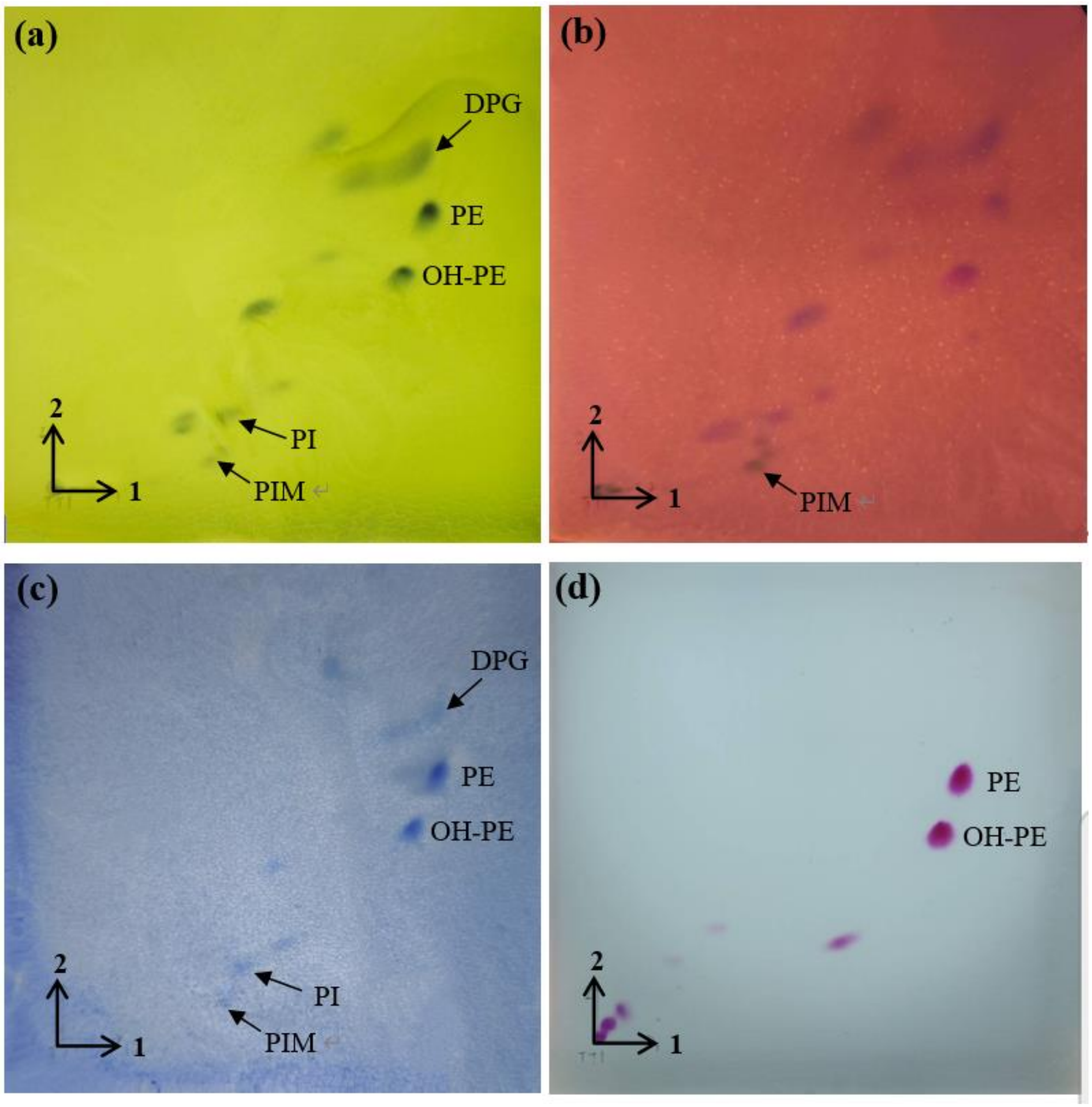

Fig. S1. Polar lipids composition of strain HUAS TT1^{T}.

Note: a, Molybdophosphoric acid (for total lipids); b, anisaldehyde (for phosphatidylinositol mannosides); c, Molybdenum blue reagent (for phospholipids); d, Ninhydrin (by baking at 90 °C for aminolipids). Chloroform/methanol/water (65:25:4, by vol.) was used in the first direction (1), followed by chloroform/acetic acid/methanol/water (80:15:12:4, by vol.) in the second direction (2).

Abbreviations: DPG, diphosphatidylglycerol; OH-PE, hydroxy phosphatidylethanolamine; PE, phosphatidylethanolamine; PI, phosphatidylinositol; PIM, phosphatidylinositol mannosides.

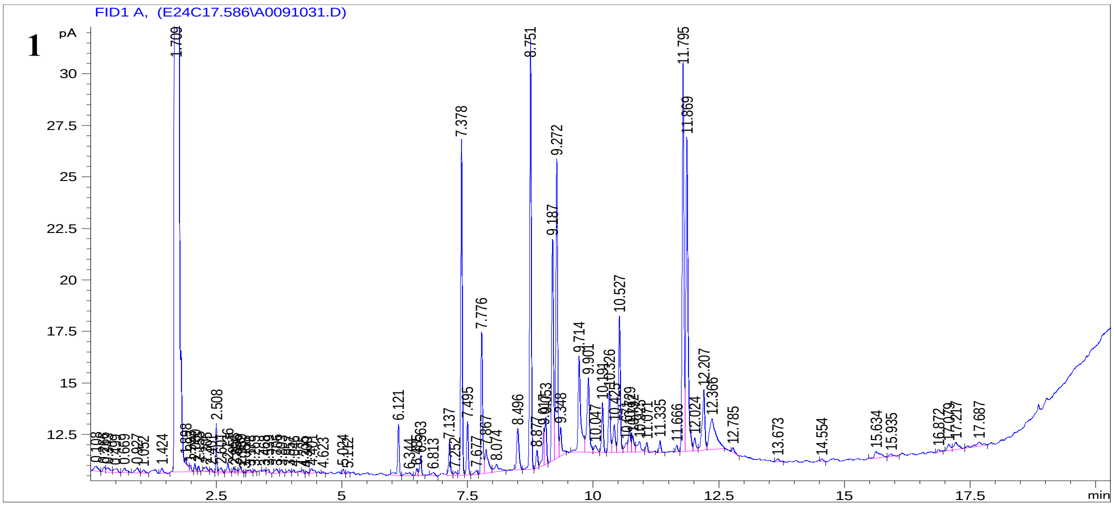
1
FID1 A, (E24C17.586\A0091031.D)
pA
min
1.709
7.378
8.751
9.272
9.187
7.776
10.527
11.795
11.869
12.207
6.121

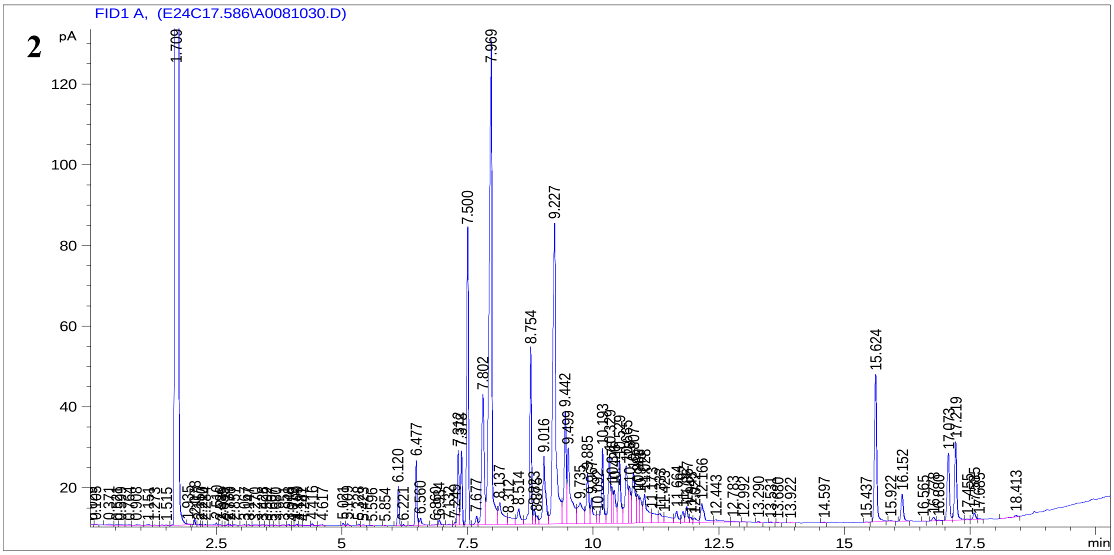
2
FID1 A, (E24C17.586\A0081030.D)
pA
min
1.709
7.969
7.500
9.227
8.754
7.802
9.442
15.624
17.219
6.477

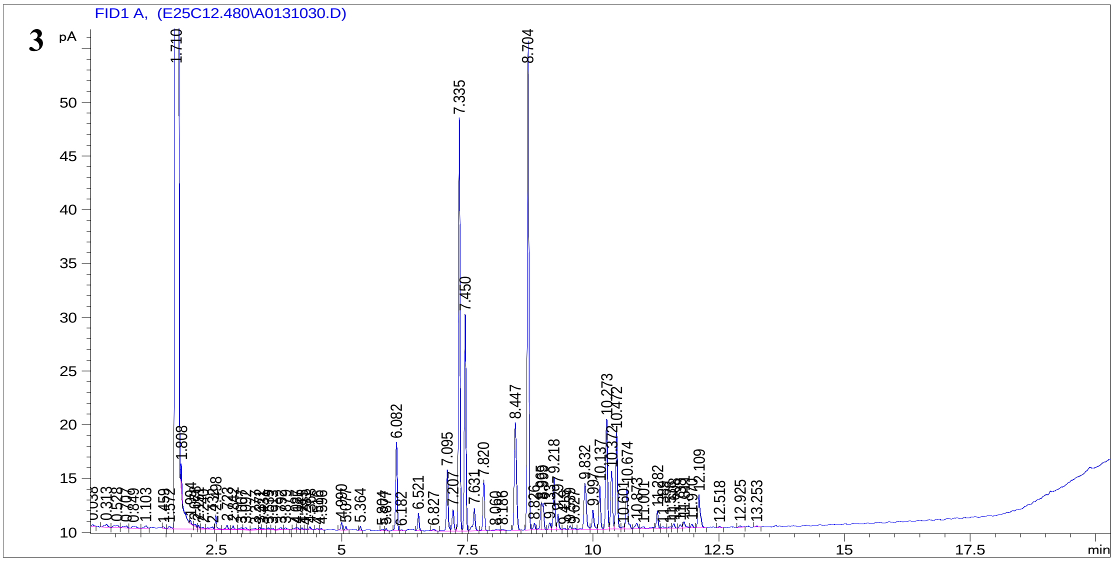
3
FID1 A, (E25C12.480\A0131030.D)
pA
min
1.710
8.704
7.335
7.450
8.447
6.082
10.273
10.472
1.808
12.109

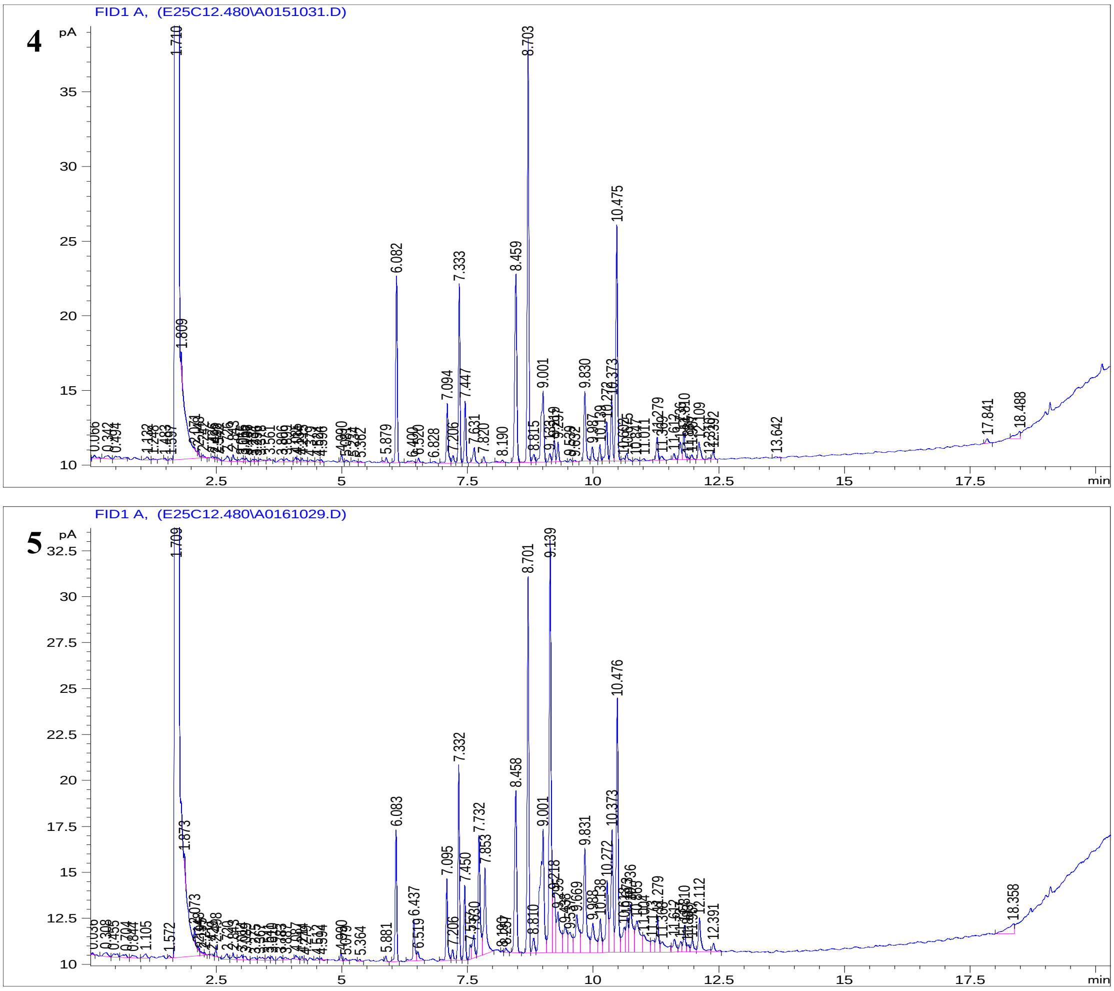

Fig. S2. The MIDI chromatogram of strain HUAS TT1^{T}.

Note: 1, HUAS TT1^{T}; 2, *S. hoggarensis* CCUG 60214^{T}; 3, *S. saharensis* DSM 45456^{T}; 4, *S. yanglingensis* Hhs.015^{T}; 5, *S. longispora* JCM 3314^{T}.

1

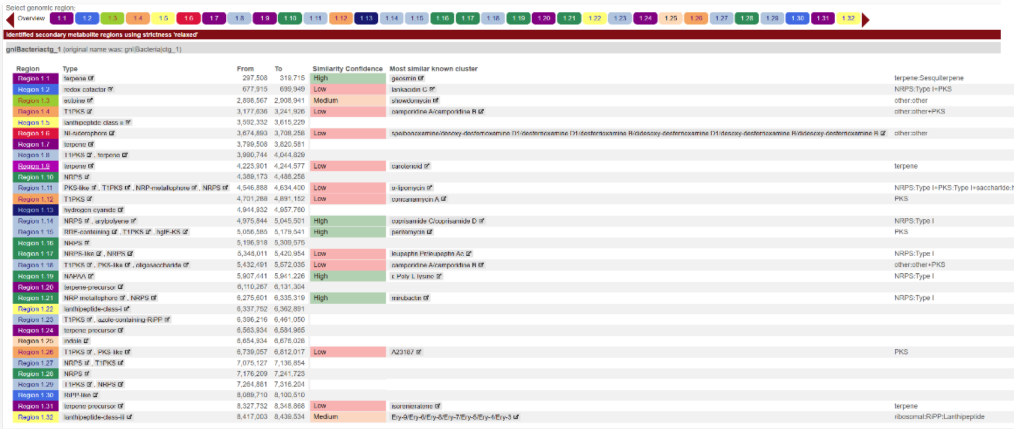

Select genomic region:

Overview 1.1 1.2 1.3 1.4 1.5 1.6 1.7 1.8 1.9 1.10 1.11 1.12 1.13 1.14 1.15 1.16 1.17 1.18 1.19 1.20 1.21 1.22 1.23 1.24 1.25 1.26 1.27 1.28 1.29 1.30 1.31 1.32

Identified secondary metabolite regions using strictness 'relaxed'

gnlBacteriactg_1 (original name was: gnl|Bacteria|ctg_1)

Region	Type	From	To	Similarity Confidence	Most similar known cluster	
Region 1.1	terpene	297,508	319,715	High	geosmin	terpene:Sesquiterpene
Region 1.2	redox cofactor	677,915	699,949	Low	lankacidin C	NRPS:Type I+PKS
Region 1.3	ectoine	2,898,567	2,908,941	Medium	showdomycin	other:other
Region 1.4	T1PKS	3,177,636	3,241,926	Low	camporidine A/camporidine B	other:other+PKS
Region 1.5	lanthipeptide class ii	3,592,332	3,615,229			
Region 1.6	NI-siderophore	3,674,893	3,708,258	Low	spalbonoxamine/desoxy-desferrioxamine D1/desferrioxamine D1/desferrioxamine B/didesoxy-desferrioxamine D1/desoxy-desferrioxamine B/didesoxy-desferrioxamine B	other:other
Region 1.7	terpene	3,799,508	3,820,581			
Region 1.8	T1PKS , terpene	3,990,744	4,044,829			
Region 1.9	terpene	4,223,901	4,244,577	Low	carotenoid	terpene
Region 1.10	NRPS	4,389,173	4,488,258			
Region 1.11	PKS-like , T1PKS , NRP-metallophore , NRPS	4,546,888	4,634,400	Low	u-lipomycin	NRPS:Type I+PKS:Type I+saccharide:hybrid/tailoring
Region 1.12	T1PKS	4,701,288	4,891,152	Low	concanamycin A	PKS
Region 1.13	hydrogen cyanide	4,944,932	4,957,760			
Region 1.14	NRPS , arylpolyene	4,975,844	5,045,501	High	coprisamide C/coprisamide D	NRPS:Type I
Region 1.15	RRE-containing , T1PKS , hglE-KS	5,056,585	5,179,541	High	pentamycin	PKS
Region 1.16	NRPS	5,196,918	5,309,575			
Region 1.17	NRPS-like , NRPS	5,348,011	5,420,954	Low	leupeptin Pr/leupeptin Ac	NRPS:Type I
Region 1.18	T1PKS , PKS-like , oligosaccharide	5,432,491	5,572,035	Low	camporidine A/camporidine B	other:other+PKS
Region 1.19	NAPAA	5,907,441	5,941,226	High	ε Poly L lysine	NRPS:Type I
Region 1.20	terpene-precursor	6,110,267	6,131,304			
Region 1.21	NRP metallophore , NRPS	6,275,601	6,335,319	High	mirubactin	NRPS:Type I
Region 1.22	lanthipeptide-class-i	6,337,752	6,362,891			
Region 1.23	T1PKS , azole-containing-RiPP	6,396,216	6,461,050			
Region 1.24	terpene precursor	6,563,934	6,584,965			
Region 1.25	indole	6,654,934	6,678,028			
Region 1.26	T1PKS , PKS-like	6,739,057	6,812,017	Low	A23187	PKS
Region 1.27	NRPS , T1PKS	7,075,127	7,136,854			
Region 1.28	NRPS	7,176,209	7,241,723			
Region 1.29	T1PKS , NRPS	7,264,881	7,316,204			
Region 1.30	RiPP-like	8,089,710	8,100,510			
Region 1.31	terpene precursor	8,327,732	8,348,868	Low	isorenieratene	terpene
Region 1.32	lanthipeptide-class-iii	8,417,003	8,439,534	Medium	Ery-9/Ery-6/Ery-8/Ery-7/Ery-5/Ery-4/Ery-3	ribosomal:RiPP:Lanthipeptide

2

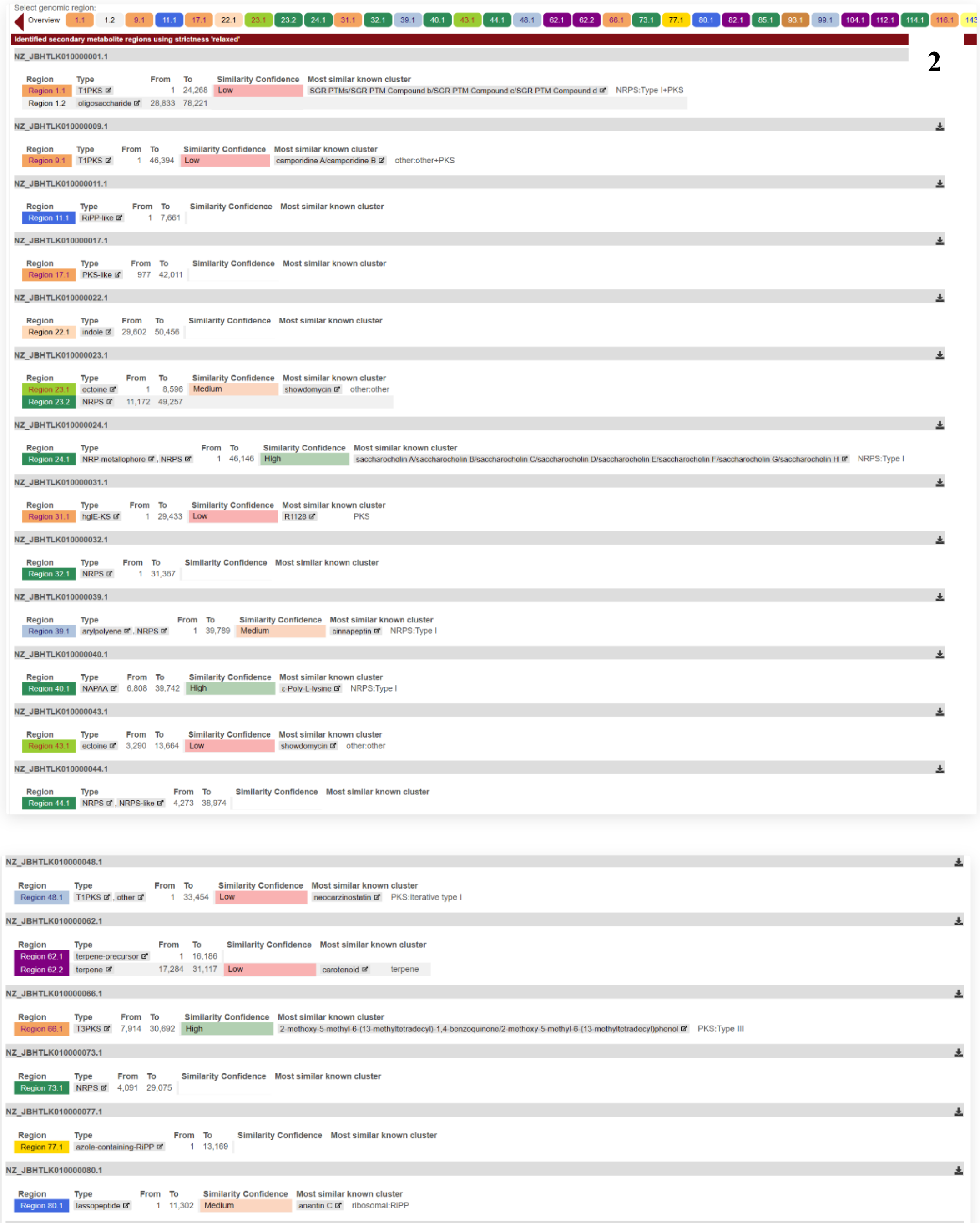

Select genomic region:

Overview 1.1 1.2 9.1 11.1 17.1 22.1 23.1 23.2 24.1 31.1 32.1 39.1 40.1 43.1 44.1 48.1 62.1 62.2 66.1 73.1 77.1 80.1 82.1 85.1 93.1 99.1 104.1 112.1 114.1 116.1 143

Identified secondary metabolite regions using strictness 'relaxed'

NZ_JBHTLK010000001.1

Region	Type	From	To	Similarity Confidence	Most similar known cluster	
Region 1.1	T1PKS	1	24,268	Low	SGR PTMs/SGR PTM Compound b/SGR PTM Compound c/SGR PTM Compound d	NRPS:Type I+PKS
Region 1.2	oligosaccharide	28,833	78,221			

NZ_JBHTLK010000009.1

Region	Type	From	To	Similarity Confidence	Most similar known cluster	
Region 9.1	T1PKS	1	46,394	Low	camporidine A/camporidine B	other:other+PKS

NZ_JBHTLK010000011.1

Region	Type	From	To	Similarity Confidence	Most similar known cluster
Region 11.1	RiPP-like	1	7,661		

NZ_JBHTLK010000017.1

Region	Type	From	To	Similarity Confidence	Most similar known cluster
Region 17.1	PKS-like	977	42,011		

NZ_JBHTLK010000022.1

Region	Type	From	To	Similarity Confidence	Most similar known cluster
Region 22.1	indole	29,602	50,456		

NZ_JBHTLK010000023.1

Region	Type	From	To	Similarity Confidence	Most similar known cluster	
Region 23.1	ectoine	1	8,596	Medium	showdomycin	other:other
Region 23.2	NRPS	11,172	49,257			

NZ_JBHTLK010000024.1

Region	Type	From	To	Similarity Confidence	Most similar known cluster	
Region 24.1	NRP metallophore , NRPS	1	46,146	High	saccharocholin A/saccharocholin B/saccharocholin C/saccharocholin D/saccharocholin E/saccharocholin F/saccharocholin G/saccharocholin H	NRPS:Type I

NZ_JBHTLK010000031.1

Region	Type	From	To	Similarity Confidence	Most similar known cluster	
Region 31.1	hglE-KS	1	29,433	Low	R1128	PKS

NZ_JBHTLK010000032.1

Region	Type	From	To	Similarity Confidence	Most similar known cluster
Region 32.1	NRPS	1	31,367		

NZ_JBHTLK010000039.1

Region	Type	From	To	Similarity Confidence	Most similar known cluster	
Region 39.1	arylpolyene , NRPS	1	39,789	Medium	cinnapeptin	NRPS:Type I

NZ_JBHTLK010000040.1

Region	Type	From	To	Similarity Confidence	Most similar known cluster	
Region 40.1	NAPAA	6,808	39,742	High	ε Poly L lysine	NRPS:Type I

NZ_JBHTLK010000043.1

Region	Type	From	To	Similarity Confidence	Most similar known cluster	
Region 43.1	ectoine	3,290	13,664	Low	showdomycin	other:other

NZ_JBHTLK010000044.1

Region	Type	From	To	Similarity Confidence	Most similar known cluster
Region 44.1	NRPS , NRPS-like	4,273	38,974		

NZ_JBHTLK010000048.1

Region	Type	From	To	Similarity Confidence	Most similar known cluster	
Region 48.1	T1PKS , other	1	33,454	Low	neocarzinostatin	PKS:Iterative type I

NZ_JBHTLK010000062.1

Region	Type	From	To	Similarity Confidence	Most similar known cluster	
Region 62.1	terpene-precursor	1	16,186			
Region 62.2	terpene	17,284	31,117	Low	carotenoid	terpene

NZ_JBHTLK010000066.1

Region	Type	From	To	Similarity Confidence	Most similar known cluster	
Region 66.1	T3PKS	7,914	30,692	High	2-methoxy-5-methyl-6-(13-methyltetradecyl)-1,4-benzoquinone/2-methoxy-5-methyl-6-(13-methyltetradecyl)phenol	PKS:Type III

NZ_JBHTLK010000073.1

Region	Type	From	To	Similarity Confidence	Most similar known cluster
Region 73.1	NRPS	4,091	29,075		

NZ_JBHTLK010000077.1

Region	Type	From	To	Similarity Confidence	Most similar known cluster
Region 77.1	azole-containing-RiPP	1	13,169		

NZ_JBHTLK010000080.1

Region	Type	From	To	Similarity Confidence	Most similar known cluster	
Region 80.1	lassopeptide	1	11,302	Medium	anantin C	ribosomal:RiPP

Record	Region	Type	From	To	Similarity Confidence	Most similar known cluster	
NZ_JBHTLK010000082.1	Region 82.1	terpene	14,411	26,734			
NZ_JBHTLK010000085.1	Region 85.1	NRPS	1	26,369	Low	cysteoamide	NRPS:Type I
NZ_JBHTLK010000093.1	Region 93.1	arylpolyene	1	25,088			
NZ_JBHTLK010000099.1	Region 99.1	NRPS , NRPS-like , transAT-PKS-like	1	23,901			
NZ_JBHTLK010000104.1	Region 104.1	terpene	7,067	23,492	Low	isorenieratene	terpene
NZ_JBHTLK010000112.1	Region 112.1	terpene	3,652	21,825	High	geosmin	terpene:Sesquiterpene
NZ_JBHTLK010000114.1	Region 114.1	NRPS-like	1	21,604			
NZ_JBHTLK010000116.1	Region 116.1	transAT-PKS	1	21,426	Low	alpiniamide	other:aminocoumarin+PKS+NRPS:Type I
NZ_JBHTLK010000143.1	Region 143.1	lanthipeptide-class-ii	5,701	17,867			
NZ_JBHTLK010000147.1	Region 147.1	betalactone	1	17,605			
NZ_JBHTLK010000159.1	Region 159.1	NRPS-like	1	16,576	High	rhizomide A/rhizomide B/rhizomide C	NRPS:Type I
NZ_JBHTLK010000177.1	Region 177.1	NRPS	1	15,073			
NZ_JBHTLK010000185.1	Region 185.1	T1PKS	1	14,629			
NZ_JBHTLK010000194.1	Region 194.1	T3PKS	1	13,953			

Record	Region	Type	From	To	Similarity Confidence	Most similar known cluster	
NZ_JBHTLK010000204.1	Region 204.1	RiPP-like	1	10,225			
NZ_JBHTLK010000205.1	Region 205.1	redox-cofactor	1	12,707	Low	lankacidin C	NRPS:Type I+PKS
NZ_JBHTLK010000207.1	Region 207.1	NRPS-like	1	12,574			
NZ_JBHTLK010000217.1	Region 217.1	NI-siderophore	1	11,618	Low	speibonoxamine/desoxy-desferrioxamine D1/desferrioxamine D1/desferrioxamine B/didesoxy-desferrioxamine D1/desoxy-desferrioxamine B/didesoxy-desferrioxamine B	other:other
NZ_JBHTLK010000240.1	Region 240.1	NRPS-like	1	10,249			
NZ_JBHTLK010000243.1	Region 243.1	NRPS	1	10,161			
NZ_JBHTLK010000245.1	Region 245.1	NRPS	1	10,057	Low	combamide	NRPS:Type I+PKS
NZ_JBHTLK010000275.1	Region 275.1	terpene-precursor	1	8,285			
NZ_JBHTLK010000292.1	Region 292.1	NRPS	1	7,360			
NZ_JBHTLK010000354.1	Region 354.1	NRPS	1	4,615			
NZ_JBHTLK010000370.1	Region 370.1	hglE-KS	1	3,990			
NZ_JBHTLK010000401.1	Region 401.1	NRPS	1	3,136			

3

Select genomic region:

Overview | 1.1 | 1.2 | 1.3 | 1.4 | 1.5 | 1.6 | 1.7 | 1.8 | 1.9 | 1.10 | 1.11 | 1.12 | 1.13 | 1.14 | 1.15 | 1.16 | 1.17 | 1.18 | 1.19 | 1.20 | 1.21 | 1.22 | 1.23 | 1.24 | 1.25 | 1.26 | 1.27 | 1.28 | 1.29 | 1.30 | 1.31 | 1.32 | 1.33 | 1.34 | 1.35 | 1.36 | 1.37 | 1.38

Identified secondary metabolite regions using strictness 'relaxed'

GCA_006716745.11VFPP01000001.1Saccharothrix (original name was: GCA_006716745.1|1|VFPP01000001.1|Saccharothrix...)

Region	Type	From	To	Similarity Confidence	Most similar known cluster	
Region 1	T1PKS, PKS-like, oligosaccharide	1	77,895	Medium	meridamycin	NRPS:Type I+PKS
Region 2	RiPP like, T1PKS, NRPS like	96,389	219,010	Low	sceliphrolactam	PKS
Region 3	melanin	222,182	232,619			
Region 4	NRPS	259,328	319,424			
Region 5	T1PKS, prodigiosin	382,278	408,355			
Region 6	NAPAA	639,716	673,504	High	ε-Poly-L-lysine	NRPS:Type I
Region 7	ectoine	1,011,613	1,021,987	Medium	showdomycin	other:other
Region 8	azole-containing-RiPP, NRPS	1,272,223	1,348,120			
Region 9	terpene-precursor	1,512,292	1,533,332			
Region 10	NRP metallophore, NRPS	1,612,766	1,690,228	High	azetidomonamide A/azetidomonamide B	NRPS:Type I
Region 11	terpene-precursor	1,997,608	2,018,696			
Region 12	PKS-like, T1PKS	2,103,799	2,151,879	Low	Hisenarsan	PKS:Type I
Region 13	T3PKS	2,300,637	2,341,704	High	naringenin	PKS:Type III
Region 14	NRPS-like	2,633,561	2,676,941	Low	lomofungin	other:other
Region 15	HR-T2PKS	2,729,187	2,771,460			
Region 16	RiPP-like	2,903,739	2,914,536			
Region 17	terpene	3,129,816	3,153,379	Low	carotenoid	terpene
Region 18	lanthipeptide class iii	3,224,837	3,247,368	Medium	Ery 9/Ery 6/Ery 8/Ery 7/Ery 5/Ery 4/Ery 3	ribosomal:RiPP:Lanthipeptide
Region 19	RiPP like	3,440,353	3,450,814			
Region 20	terpene	3,643,842	3,666,052	High	geosmin	terpene:Sesquiterpene
Region 21	RiPP-like	3,812,088	3,823,341			
Region 22	redox-cofactor	4,040,516	4,062,549	Low	lankacidin C	NRPS:Type I+PKS
Region 23	terpene-precursor	6,972,752	6,993,834			
Region 24	lanthipeptide-class-ii	7,014,777	7,037,614	Low	aborycin	ribosomal:RiPP
Region 25	NI-siderophore	7,100,752	7,134,003	Low	speibonoxamine/desoxy-desferrioxamine D1/desferrioxamine D1/desferrioxamine B/didesoxy-desferrioxamine D1/desoxy-desferrioxamine D/didesoxy-desferrioxamine B	other:other
Region 26	terpene	7,230,604	7,251,677			
Region 27	T1PKS	7,368,347	7,414,112			
Region 28	terpene-precursor	7,425,915	7,447,591			
Region 29	NRPS, NRPS like, T1PKS, RRE containing	7,483,101	7,548,082			
Region 30	indole	7,658,559	7,679,653			
Region 31	thioamide-NRP, HR-T2PKS, NRPS	7,895,575	8,029,449	Low	collbrimycin	NRPS:Type I+other:other
Region 32	T2PKS, T3PKS, terpene	8,162,114	8,262,875	Low	α naphtocyclinoic acid/fogacin	PKS
Region 33	terpene	8,469,676	8,490,959	Low	2 methylisoborneol	terpene:Monoterpene
Region 34	NRPS like	8,495,347	8,539,414			
Region 35	butyrolactone	8,569,737	8,580,663			
Region 36	terpene	8,599,047	8,620,159			
Region 37	NRPS-like, NRPS, PKS-like	8,620,367	8,715,125	Low	leupeptin Pr/leupeptin Ac	NRPS:Type I
Region 38	T1PKS	8,839,846	8,907,344	Low	argimycin PI/argimycin PII/nigrifectin/argimycin PIV/argimycin PV/argimycin PVI/argimycin PIX	PKS:Type I

4

Select genomic region:

Overview | 2.1 | 2.2 | 2.3 | 4.1 | 5.1 | 5.2 | 5.3 | 5.4 | 6.1 | 6.2 | 6.3 | 7.1 | 8.1 | 9.1 | 10.1 | 11.1 | 12.1 | 12.2 | 13.1 | 14.1 | 17.1 | 18.1 | 19.1 | 21.1 | 22.1 | 26.1 | 32.1 | 33.1 | 34.1 | 37.1 | 38.1 | 45.1 | 46.1 | 46.2 | 46.3 | 46.4 | 47.1 | 47.2 | 49.1 | 49.2 | 49.3

Identified secondary metabolite regions using strictness 'relaxed'

NZ_NSDM01000010.1

Region	Type	From	To	Similarity Confidence	Most similar known cluster	
Region 2.1	NRPS	1	24,052			
Region 2.2	T1PKS, NRPS	163,979	261,343	Low	kedarcidin	NRPS:Type I+PKS:Iterative type I
Region 2.3	terpene	305,179	327,358			

NZ_NSDM01000012.1

Region	Type	From	To	Similarity Confidence	Most similar known cluster
Region 4.1	terpene	143,325	165,535		

NZ_NSDM01000013.1

Region	Type	From	To	Similarity Confidence	Most similar known cluster	
Region 5.1	terpene	1	12,476			
Region 5.2	NI siderophore	105,298	138,562	Low	speibonoxamine/desoxy desferrioxamine D1/desferrioxamine D1/desferrioxamine B/didesoxy desferrioxamine D1/desoxy desferrioxamine D/didesoxy desferrioxamine B	other:other
Region 5.3	lanthipeptide-class-ii	177,660	200,690			
Region 5.4	terpene precursor	208,560	229,657			

NZ_NSDM01000014.1

Region	Type	From	To	Similarity Confidence	Most similar known cluster
Region 6.1	T1PKS, azoxy-crosslink	25,189	69,127		
Region 6.2	T1PKS	71,155	124,906		
Region 6.3	RiPP-like	158,825	170,012		

NZ_NSDM01000015.1

Region	Type	From	To	Similarity Confidence	Most similar known cluster
Region 7.1	NRPS like, T1PKS	100,487	149,582		

NZ_NSDM01000016.1

Region	Type	From	To	Similarity Confidence	Most similar known cluster
Region 8.1	NRPS-like, betalactone	121,285	148,281		

NZ_NSDM01000017.1

Region	Type	From	To	Similarity Confidence	Most similar known cluster
Region 9.1	T3PKS	56,558	87,607		

NZ_NSDM01000018.1

Region	Type	From	To	Similarity Confidence	Most similar known cluster	
Region 10.1	T1PKS	109,939	142,576	Low	eurocidin D/eurocidin E	NRPS:Type I+PKS:Type I

NZ_NSDM01000019.1

Region	Type	From	To	Similarity Confidence	Most similar known cluster
Region 11.1	NRPS-like	1	21,568		

NZ_NSDM01000002.1

Region	Type	From	To	Similarity Confidence	Most similar known cluster	
Region 12.1	acyl_amino_acids, NRPS, butyrolactone, T1PKS, PKS-like, transAT-PKS, NRPS-like, hglE-KS	5,715	349,406	High	macrotermycins	PKS
Region 12.2	NRPS like, T1PKS, T2PKS	400,840	497,629			

NZ_NSDM01000020.1

Region	Type	From	To	Similarity Confidence	Most similar known cluster
Region 13.1	RiPP like	76,698	87,528		

NZ_NSDM01000021.1

Region	Type	From	To	Similarity Confidence	Most similar known cluster	
Region 14.1	T1PKS	1	63,771	Low	geldanamycin	PKS

NZ_NSDM01000024.1

Region	Type	From	To	Similarity Confidence	Most similar known cluster	
Region 17.1	NAPAA	14,609	48,409	High	ε-Poly-L-lysine	NRPS:Type I

NZ_NSDM01000025.1

Region	Type	From	To	Similarity Confidence	Most similar known cluster	
Region 18.1	T1PKS, hglE-KS	1	71,881	High	pentamycin	PKS

NZ_NSDM01000026.1

Region	Type	From	To	Similarity Confidence	Most similar known cluster
Region 19.1	NRPS, T1PKS	1	48,991		

NZ_NSDM01000028.1

Region	Type	From	To	Similarity Confidence	Most similar known cluster
Region 21.1	terpene	1	15,866		

NZ_NSDM01000029.1

Region	Type	From	To	Similarity Confidence	Most similar known cluster
Region 22.1	T1PKS	1	44,987		

NZ_NSDM01000032.1

Region	Type	From	To	Similarity Confidence	Most similar known cluster	
Region 26.1	NRPS	1	37,021	Low	cadaside A/cadaside B	NRPS:Type I

NZ_NSDM01000038.1

Region	Type	From	To	Similarity Confidence	Most similar known cluster
Region 32.1	T1PKS	1	4,896		

NZ_NSDM01000039.1

Region	Type	From	To	Similarity Confidence	Most similar known cluster
Region 33.1	T1PKS	1	4,631		

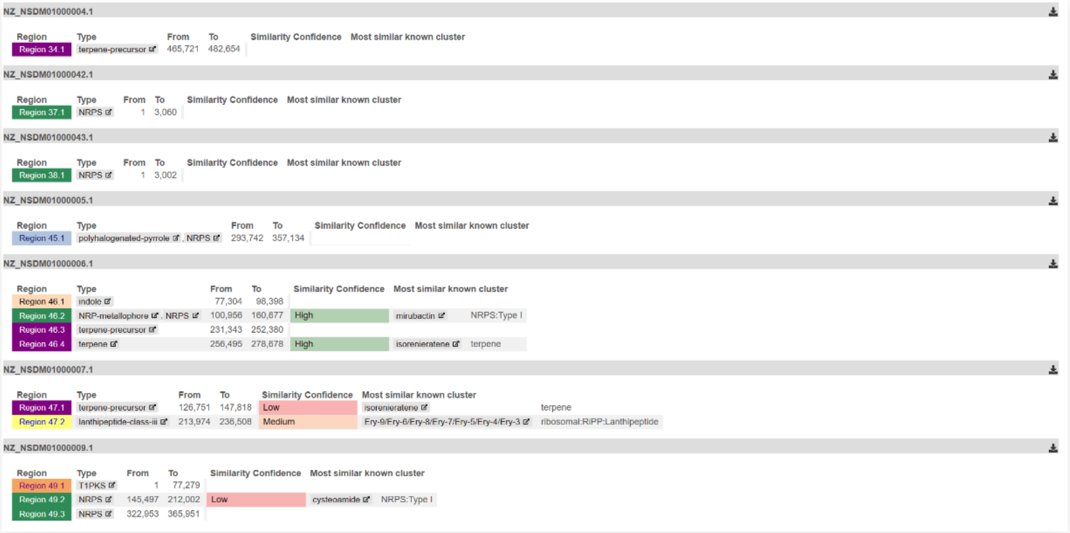

NZ_NSDM01000004.1

Region	Type	From	To	Similarity Confidence	Most similar known cluster
Region 34.1	terpene-precursor	465,721	482,654		

NZ_NSDM01000042.1

Region	Type	From	To	Similarity Confidence	Most similar known cluster
Region 37.1	NRPS	1	3,060		

NZ_NSDM01000043.1

Region	Type	From	To	Similarity Confidence	Most similar known cluster
Region 38.1	NRPS	1	3,002		

NZ_NSDM01000005.1

Region	Type	From	To	Similarity Confidence	Most similar known cluster
Region 45.1	polyhalogenated-pyrrole , NRPS	293,742	357,134		

NZ_NSDM01000006.1

Region	Type	From	To	Similarity Confidence	Most similar known cluster	
Region 46.1	indole	77,304	98,398			
Region 46.2	NRP-metallophore , NRPS	100,956	160,677	High	mirubactin	NRPS:Type I
Region 46.3	terpene-precursor	231,343	252,380			
Region 46.4	terpene	256,495	278,678	High	isorenieratene	terpene

NZ_NSDM01000007.1

Region	Type	From	To	Similarity Confidence	Most similar known cluster	
Region 47.1	terpene-precursor	126,751	147,818	Low	isorenieratene	terpene
Region 47.2	lanthipeptide-class-iii	213,974	236,508	Medium	Fry-9/Fry-6/Fry-8/Fry-7/Fry-5/Fry-4/Fry-3	ribosomal:RiPP:Lanthipeptide

NZ_NSDM01000009.1

Region	Type	From	To	Similarity Confidence	Most similar known cluster	
Region 49.1	T1PKS	1	77,279			
Region 49.2	NRPS	145,497	212,002	Low	cysteoamide	NRPS:Type I
Region 49.3	NRPS	322,953	365,951			

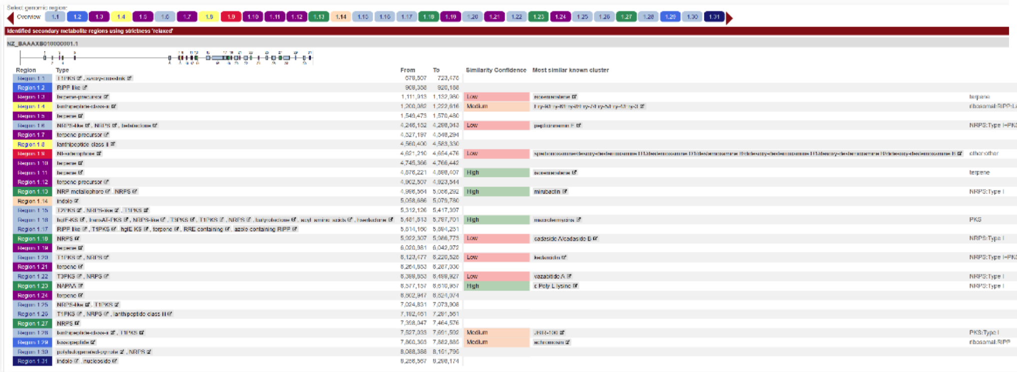

5

Fig. S3. The distribution of biosynthetic gene clusters in the genome of strains HUAS TT1^{T}, *S. hoggarensis* CCUG 60214^{T}, *S. saharensis* DSM 45456^{T}, *S. yanglingensis* Hhs.015^{T} and *S. longispora* by antiSMASH analyses.

Note: 1, HUAS TT1^{T}; 2, *S. hoggarensis* CCUG 60214^{T}; 3, *S. saharensis* DSM 45456^{T}; 4, *S. yanglingensis* Hhs.015^{T}; 5, *S. longispora* JCM 3314^{T}.

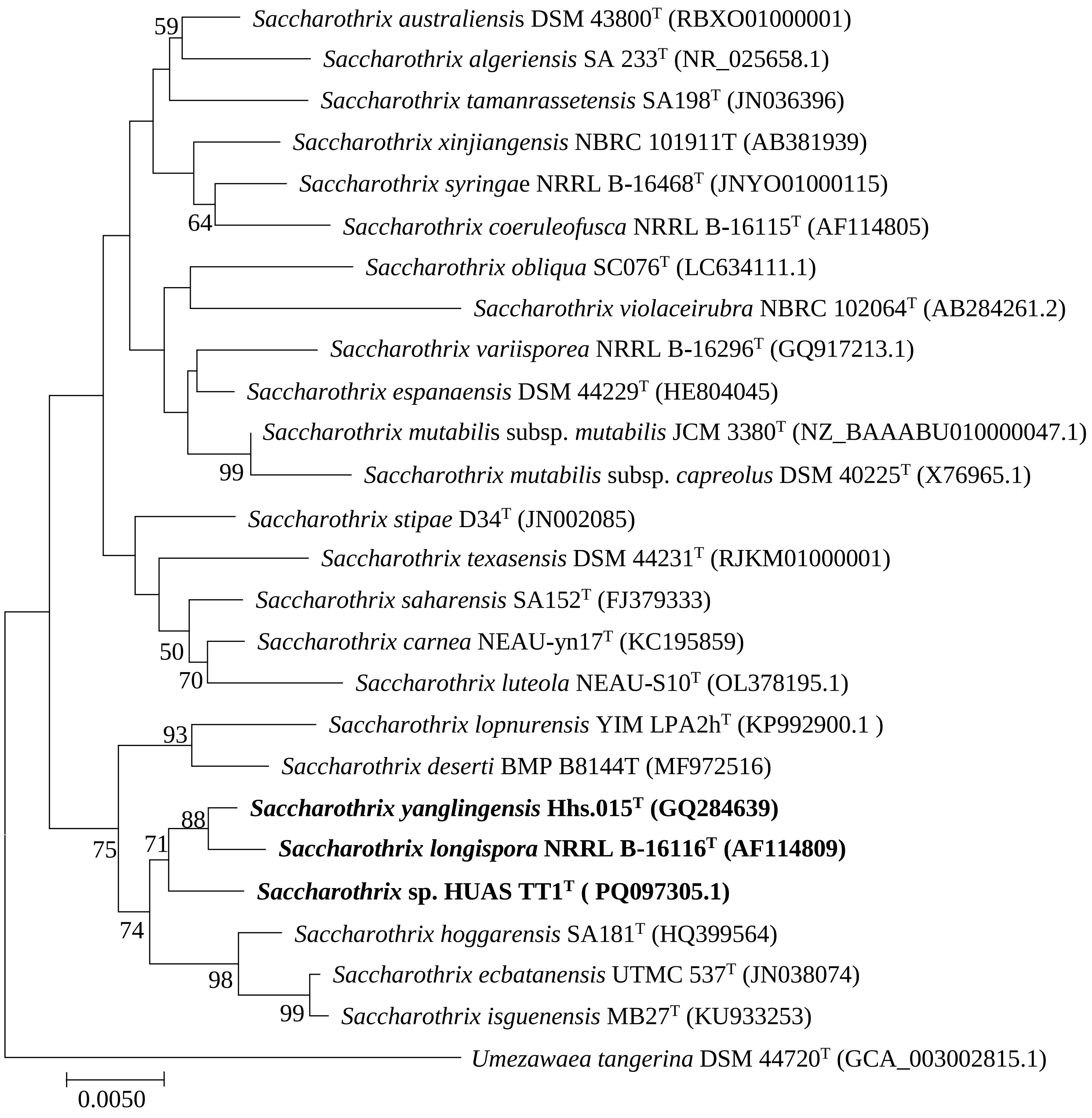

Fig. S4. Neighbour-joining phylogenetic tree based on 16S rRNA gene sequences showing the relationship between selected species of the genus *Saccharothrix*. *Mycobacterium tuberculosis* H37Rv^{T} was used as an outgroup. Bootstrap percentages over 50% derived from 1000 replications are showed at the nodes. Bar, 0.0100 nucleotide substitutions per site.

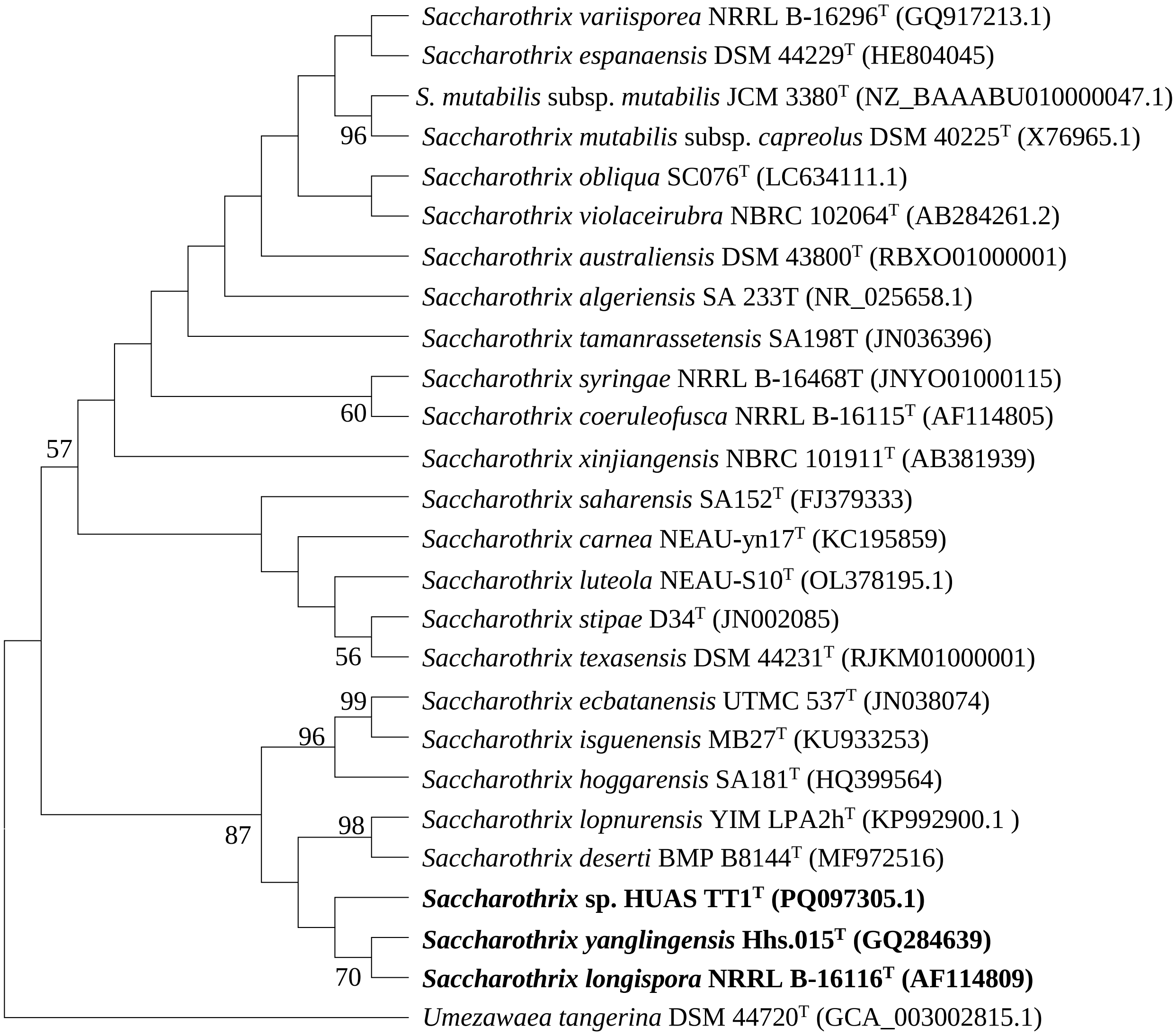

Fig. S5. Maximum-parsimony phylogenetic tree based on 16S rRNA gene sequences showing the relationship between selected species of the genus *Saccharothrix*. *Mycobacterium tuberculosis* H37Rv^{T} was used as an outgroup. Bootstrap percentages over 50% derived from 1000 replications are showed at the nodes. Bar represents 10.0.

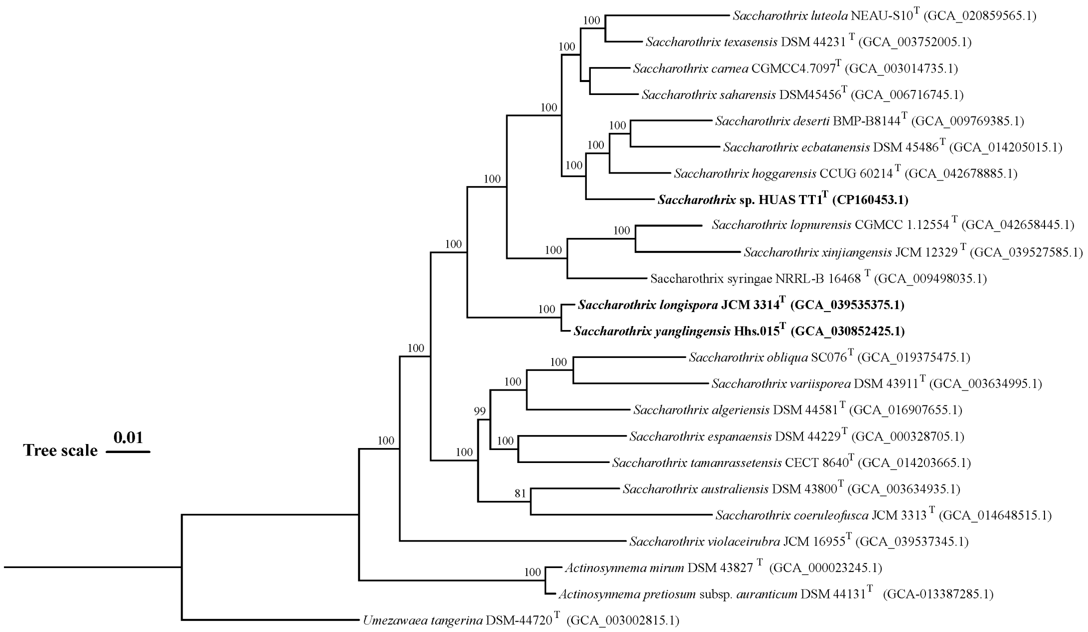

Fig. S6. EasyCG Tree of strain HUAS TT1^{T} and related reference strains were constructed. The tree was rooted at the outgroup *Umezawaea tangerina* DSM 44720^{T}. Bar represents 0.01.

Table S1. Quality analysis and GenBank assembly of genomes of strain HUAS TT1^T and related reference strains.

No.	Species	Strain	Size (bp)	GenBank assembly	Completeness	Contamination
1	*Saccharothrix camelliae*	HUAS TT1^T	8,515,408	CP160453.1	99.9%	1.91%
2	*Saccharothrix hoggarensis*	CCUG 60214^T	7,623,907	GCA_042678885.1	99.38%	0.66%
3	*Saccharothrix saharensis*	DSM45456^T	8,924,967	GCA_006716745.1	99.73%	1.66%
4	*Saccharothrix yanglingensis*	Hhs.015^T	8,286,323	GCA_030852425.1	100%	1.33%
5	*Saccharothrix longispora*	JCM 3314^T	8,393,791	GCA_039535375.1	98.84%	1.66%
6	*Saccharothrix xinjiangensis*	JCM 12329^T	9,999,587	GCA_039527585.1	98.51%	1.24%
7	*Saccharothrix violaceirubra*	JCM 16955^T	7,298,710	GCA_039537345.	98.51%	1.24%
8	*Saccharothrix variisporea*	DSM 43911^T	9,408,895	GCA_003634995.1	100%	0.08%
9	*Saccharothrix texasensis*	DSM 44231^T	9,178,199	GCA_003752005.1	99.9%	0.66%
10	*Saccharothrix tamanrassetensis*	CECT 8640^T	10,929,570	GCA_014203665.1	100%	0.5%
11	*Saccharothrix syringae*	NRRL B-16468^T	10,929,570	GCA_009498035.1	98.01%	3.17%
12	*Saccharothrix obliqua*	SC076^T	8,040,245	GCA_019375475.1	99.67%	1.34%
13	*Saccharothrix luteola*	NEAU-S10^T	10,305,394	GCA_020859565.1	98.23%	1.58%
14	*Saccharothrix lopnurensis*	CGMCC 1.12554^T	8,485,199	GCA_042658445.1	98.01%	1%
15	*Saccharothrix espanaensis*	DSM 44229^T	9,360,653	GCA_000328705.1	99.5%	1.12%
16	*Saccharothrix ecbatanensis*	DSM 45486^T	9,689,237	GCA_014205015.1	100%	2.26%
17	*Saccharothrix deserti*	BMP B8144^T	10,832,261	GCA_009769385.1	100%	4.98%
18	*Saccharothrix coeruleofusca*	JCM 3313^T	7,692,265	GCA_014648515.1	99.25%	1.49%
19	*Saccharothrix carnea*	CGMCC4.7097^T	8,918,169	GCA_003014735.1	100%	2.57%
20	*Saccharothrix australiensis*	DSM 43800^T	8,918,169	GCA_003634935.1	99.5%	1.58%
21	*Saccharothrix algeriensis*	DSM 44581^T	6,878,582	GCA_016907655.1	99%	1.66%
22	*Actinosynnema pretiosum* subsp. *auranticum*	DSM 44131^T	8,105,537	GCA_013387285.1	98.91%	0.1%
23	*Actinosynnema mirum*	DSM 43827^T	8,248,144	GCA_000023245.1	99.9%	0%
24	*Umezawaea tangerina*	DSM 44720^T	10,685,010	GCA_003002815.1	99.50%	1.49%

Table S2. Cultural characteristics between strain strain HUAS TT1^T, *S. hoggarensis* DSM 45457^T, *S. saharensis* DSM 45456^T, *S. yanglingensis* CGMCC 4.5627^T and *S. longispora* CGMCC 4.1357^T.

Characteristics	1	2	3	4	5
Color of aerial mycelium on No.1	White	White	Yellow	White	White
Color of substrate mycelium on No.1	Chamois	Ivory yellow	Dahlia yellow	Chamois	Ivory yellow
Difusible pigment on No.1	Olive-ocher	None	None	None	None
Color of aerial mycelium on R2A	White	Ivory yellow	Yellow	White	White
Color of substrate mycelium on R2A	Light vinaceous-cinnamon	Cream-buff	Yellow orange	Pinkish cinnamon	Pale pinkish buff
Difusible pigment on R2A	Cinnamon-buff	None	None	None	None
Color of aerial mycelium on ISP 2	White	White	Bluish-white	Yellow	Yellow
Color of substrate mycelium on ISP 2	Pale cinnamon-pink	Pinkish Buff	Brown	Yellow orange	Yellow orange
Difusible pigment on ISP 2	Pale vinaceous-fawn	None	Deep bluish	None	None
Color of aerial mycelium on ISP 3	White	Ivory yellow	Light bluish-grey	White	White
Color of substrate mycelium on ISP 3	Ivory yellow	Chamois	Yellowish-brown	Ivory yellow	Ivory yellow
Difusible pigment on ISP 3	Pale olive-buff	Cream-buff	None	None	None
Color of aerial mycelium on ISP 4	White	White	Greyish	Marguerite yellow	White
Color of substrate mycelium on ISP 4	Tilleul-buff	Ivory yellow	Greyish	Pinkish cinnamon	White
Difusible pigment on ISP 4	Pale drab-gray	None	Bluish	None	None
Color of aerial mycelium on ISP 5	White	White	Yellow	White	White
Color of substrate mycelium on ISP 5	Pale pinkish buff	Pale pinkish cinnamon	Shell pink	Pinkish cinnamon	Pale pinkish buff
Difusible pigment on ISP 5	Pale ecru-drab	None	None	None	None
Color of aerial mycelium on ISP 6	White	Ivory yellow	Yellow	Ivory yellow	Ivory yellow
Color of substrate mycelium on ISP 6	Pale grayish vinaceous	Pinkish Buff	Yellow	Chamois	Pale pinkish buff

Difusible pigment on ISP 6	Ecru-drab	None	None	None	None
Color of aerial mycelium on ISP 7	White	White	Yellow	White	White
Color of substrate mycelium on ISP 7	Sea-foam yellow	Ivory yellow	Tilleul-buff	Olive-ocher	White
Difusible pigment on ISP 7	Marguerite yellow	None	None	None	None

Note: 1, HUAS TT1^{T}; 2, *S. hoggarensis* DSM 45457^{T}; 3, *S. saharensis* DSM 45456^{T}; 4, *S. yanglingensis* CGMCC 4.5627^{T}; 5, *S. longispora* CGMCC 4.1357^{T}. No.1, Gause's synthetic No.1 medium; R2A, Reasoner'2A. All data were from this study.

Table S3. The peak table of strain HUAS TT1^T, *S. hoggarensis* DSM 45457^T, *S. saharensis* DSM 45456^T, *S. yanglingensis* CGMCC 4.5627^T and *S. longispora* CGMCC 4.1357^T.

HUAS TT1^T								
RT	Response	Ar/Ht	RFact	ECL	Peak Name	Percent	Comment1	Comment2
0.108	2731	0.074	----	3.318		----	< min rt	
0.266	1635	0.050	----	3.681		----	< min rt	
0.343	2113	0.067	----	3.857		----	< min rt	
0.499	1975	0.068	----	4.214		----	< min rt	
0.669	1727	0.058	----	4.601		----	< min rt	
0.927	2361	0.063	----	5.191		----	< min rt	
1.052	1165	0.054	----	5.479		----	< min rt	
1.424	1515	0.044	----	6.329		----	< min rt	
1.709	4.748E+8	0.026	----	6.980	SOLVENT PEAK	----	< min rt	
1.898	916	0.025	----	7.426		----	< min rt	
1.993	776	0.033	----	7.644		----	< min rt	
2.080	1725	0.034	----	7.841		----	< min rt	
2.155	939	0.028	----	8.013		----	< min rt	
2.305	1957	0.064	----	8.354		----	< min rt	
2.401	1074	0.045	----	8.573		----	< min rt	
2.508	6889	0.023	----	8.815		----	< min rt	
2.601	2037	0.052	----	9.028		----		
2.736	2717	0.038	----	9.336		----		
2.853	992	0.028	1.125	9.603	10:0 iso	0.18	ECL deviates -	Reference -0.005
2.896	865	0.037	----	9.701		----		
2.989	1633	0.049	----	9.913		----		
3.068	96	0.016	----	10.069		----	< min ar/ht	
3.178	1426	0.064	----	10.251		----		
3.265	562	0.032	1.086	10.396	9:0 3OH	0.10	ECL deviates -	
3.468	530	0.044	----	10.734		----		
3.539	998	0.047	----	10.852		----		
3.706	1715	0.056	----	11.099		----		
3.803	977	0.043	----	11.222		----		
3.957	898	0.046	1.044	11.416	10:0 3OH	0.15	ECL deviates -	
4.046	455	0.029	----	11.530		----		
4.235	726	0.033	----	11.768		----		
4.300	578	0.035	----	11.851		----		
4.401	1893	0.062	----	11.978		----		
4.623	460	0.038	----	12.209		----		
5.024	998	0.032	1.005	12.615	13:0 iso	0.16	ECL deviates	Reference 0.003
5.112	452	0.037	1.002	12.705	13:0 anteiso	0.07	ECL deviates	Reference 0.005
6.121	11862	0.037	0.980	13.618	14:0 iso	1.86	ECL deviates -	Reference 0.000
6.344	2410	0.098	----	13.809		----	> max ar/ht	
6.485	1791	0.038	----	13.931		----		
6.563	4515	0.037	0.972	13.998	14:0	0.70	ECL deviates -	Reference 0.000
6.813	1410	0.061	0.969	14.190	13:0 2OH	0.22	ECL deviates -	
7.137	8143	0.038	0.965	14.438	15:1 iso G	1.25	ECL deviates -	
7.252	1366	0.051	0.964	14.526	15:1 anteiso A	0.21	ECL deviates -	
7.378	71971	0.035	0.962	14.623	15:0 iso	11.06	ECL deviates	Reference 0.002
7.495	11788	0.035	0.961	14.712	15:0 anteiso	1.81	ECL deviates -	Reference 0.001

RT	Response	Ar/Ht	RFact	ECL	Peak Name	Percent	Comment1	Comment2
7.677	1589	0.057	0.959	14.852	15:1 w6c	0.24	ECL deviates -	
7.776	32865	0.037	----	14.928		----		
7.867	10935	0.062	0.957	14.998	15:0	----	ECL deviates -	
8.074	4735	0.083	----	15.145		----	> max ar/ht	
8.496	13295	0.051	0.953	15.445	16:1 iso G	2.02	ECL deviates	
8.751	96780	0.036	0.951	15.627	16:0 iso	14.70	ECL deviates	Reference 0.001
8.877	4206	0.038	0.950	15.717	16:0 anteiso	0.64	ECL deviates -	
9.017	8642	0.036	0.950	15.816	Sum In Feature 3	1.31	ECL deviates -	16:1 w7c/16:1 w6c
9.053	11315	0.037	0.949	15.842	Sum In Feature 3	1.72	ECL deviates -	16:1 w6c/16:1 w7c
9.187	54478	0.039	----	15.937		----		
9.272	74260	0.040	0.949	15.998	16:0	11.25	ECL deviates -	Reference -0.002
9.348	8393	0.044	----	16.050		----		
9.714	34049	0.053	----	16.302		----		
9.901	26497	0.057	0.947	16.430	Sum In Feature 9	4.01	ECL deviates -	16:0 10-methyl
10.047	2797	0.055	0.946	16.531	17:1 anteiso w9c	0.42	ECL deviates	
10.191	11869	0.039	0.946	16.630	17:0 iso	1.79	ECL deviates	Reference -0.001
10.326	15353	0.039	0.946	16.723	17:0 anteiso	2.32	ECL deviates	Reference -0.001
10.425	9391	0.050	0.946	16.791	17:1 w8c	1.42	ECL deviates -	
10.527	34779	0.040	0.946	16.861	17:1 w6c	5.26	ECL deviates	
10.671	3506	0.056	----	16.960		----		
10.729	7401	0.047	0.946	17.000	17:0	1.12	ECL deviates	Reference -0.001
10.792	5249	0.045	0.946	17.043	16:1 2OH	0.79	ECL deviates -	
10.925	5675	0.068	----	17.132		----		
11.071	2232	0.040	0.946	17.231	16:0 2OH	0.34	ECL deviates -	
11.335	2796	0.037	0.946	17.410	17:0 10-methyl	0.42	ECL deviates	
11.666	1629	0.039	0.947	17.634	18:0 iso	0.25	ECL deviates	Reference -0.001
11.795	96317	0.040	0.947	17.721	Sum In Feature 5	14.58	ECL deviates	18:2 w6,9c/18:0
11.869	92372	0.044	0.948	17.771	18:1 w9c	13.98	ECL deviates	
12.024	3949	0.044	----	17.877		----		
12.207	21358	0.050	0.949	18.001	18:0	3.24	ECL deviates	Reference -0.003
12.366	27087	0.111	----	18.109		----	> max ar/ht	
12.785	1496	0.045	0.951	18.394	18:0 10-methyl,	0.23	ECL deviates	
13.673	1202	0.054	0.957	18.999	19:0	0.18	ECL deviates -	Reference -0.008
14.554	1013	0.053	----	19.606		----		
15.634	5411	0.106	----	20.351		----	> max rt	
15.935	846	0.049	----	20.559		----	> max rt	
16.872	961	0.050	----	21.205		----	> max rt	
17.079	2602	0.058	----	21.348		----	> max rt	
17.217	4402	0.076	----	21.430		----	> max rt	
17.687	4031	0.126	----	21.753		----	> max rt	
----	19957	---	----	----	Summed Feature 3	3.03	16:1 w7c/16:1 w6c	16:1 w6c/16:1 w7c
----	96317	---	----	----	Summed Feature 5	14.58	18:2 w6,9c/18:0	18:0 ante/18:2
----	26497	---	----	----	Summed Feature 9	4.01	17:1 iso w9c	16:0 10-methyl

S. hoggarensis DSM 45457^{T}								
RT	Response	Ar/Ht	RFact	ECL	Peak Name	Percent	Comment1	Comment2
0.029	241	0.018	----	3.144		----	< min rt	
0.105	613	0.034	----	3.317		----	< min rt	
0.371	2422	0.070	----	3.926		----	< min rt	
0.521	1311	0.049	----	4.268		----	< min rt	
0.599	2253	0.066	----	4.446		----	< min rt	
0.764	1849	0.073	----	4.825		----	< min rt	
0.908	2184	0.073	----	5.154		----	< min rt	
1.151	2567	0.064	----	5.708		----	< min rt	
1.273	1007	0.053	----	5.986		----	< min rt	
1.515	2162	0.086	----	6.553		----	< min rt	
1.709	4.735E+8	0.025	----	6.994	SOLVENT PEAK	----	< min rt	
1.935	206	0.025	----	7.509		----	< min rt	
2.078	4014	0.021	----	7.836		----	< min rt	
2.157	1140	0.030	----	8.016		----	< min rt	
2.210	543	0.023	----	8.136		----	< min rt	
2.284	1220	0.038	----	8.305		----	< min rt	
2.510	4870	0.047	----	8.820		----	< min rt	
2.586	1476	0.051	1.159	8.994	9:0	0.10	ECL	Reference -0.011
2.635	237	0.023	----	9.106		----		
2.737	1766	0.033	----	9.339		----		
2.770	1271	0.030	----	9.413		----		
2.857	1615	0.037	1.125	9.612	10:0 iso	0.11	ECL	Reference 0.004
3.067	1512	0.049	----	10.068		----		
3.147	1543	0.045	----	10.200		----		
3.270	2838	0.083	1.086	10.406	9:0 3OH	----	> max	
3.428	2155	0.054	----	10.668		----		
3.502	1395	0.047	----	10.792		----		
3.607	1733	0.053	----	10.966		----		
3.680	1058	0.045	----	11.067		----		
3.851	2214	0.063	----	11.283		----		
3.948	1899	0.048	----	11.406		----		
4.019	403	0.032	----	11.496		----		
4.109	1893	0.033	1.037	11.610	12:0 iso	0.12	ECL	Reference 0.000
4.181	1289	0.050	1.034	11.700	12:0 anteiso	0.08	ECL	Reference 0.001
4.247	1500	0.072	----	11.784		----		
4.416	2680	0.041	1.023	11.998	12:0	0.16	ECL	Reference -0.002
4.617	1851	0.059	----	12.203		----		
5.021	2699	0.033	1.005	12.613	13:0 iso	0.16	ECL	Reference 0.000
5.109	2126	0.033	1.002	12.703	13:0 anteiso	0.13	ECL	Reference 0.001
5.328	1486	0.032	0.996	12.926	13:1 at 12-13	0.09	ECL	
5.423	1095	0.041	----	13.018		----		
5.596	324	0.029	0.990	13.167	12:0 2OH	0.02	ECL	
5.854	781	0.049	0.985	13.389	14:1 iso E	0.05	ECL	
6.120	39557	0.032	0.980	13.618	14:0 iso	2.33	ECL	Reference 0.000
6.221	1457	0.033	0.978	13.705	14:0 anteiso	0.09	ECL	Reference -0.001
6.477	74517	0.036	----	13.925		----		
6.560	11144	0.045	0.972	13.996	14:0	0.65	ECL	Reference -0.003
6.860	1282	0.054	----	14.226		----		
6.934	5552	0.034	----	14.283		----		

7.132	1211	0.048	0.965	14.435	15:1 iso G	0.07	ECL	
7.249	2652	0.029	0.964	14.524	15:1 anteiso A	0.15	ECL	
7.312	82505	0.034	----	14.573		----		
7.378	80616	0.034	0.962	14.623	15:0 iso	4.67	ECL	Reference 0.001
7.500	330221	0.035	0.961	14.717	15:0 anteiso	19.09	ECL	Reference 0.005
7.677	11678	0.040	0.959	14.852	15:1 w6c	0.67	ECL	
7.802	230894	0.055	0.958	14.949	unknown 14.959	----	ECL	
7.969	930639	0.051	----	15.071		----	ECL	
8.137	69428	0.083	----	15.191		----	> max	
8.312	29646	0.100	----	15.315		----	> max	
8.514	37409	0.067	0.952	15.459	16:1 iso H	2.14	ECL	
8.754	227727	0.039	0.951	15.629	16:0 iso	13.03	ECL	Reference 0.003
8.823	19399	0.038	0.951	15.678	unknown 15.669	----	ECL	
8.875	12067	0.041	0.950	15.715	16:0 anteiso	0.69	ECL	
9.016	135281	0.056	0.950	15.815	Sum In Feature 3	7.73	ECL	16:1 w7c/16:1 w6c
9.227	654952	0.059	----	15.966		----	ECL	
9.442	160954	0.043	----	16.114		----		
9.499	177051	0.064	----	16.154		----		
9.735	80180	0.098	----	16.316		----	> max	
9.885	90050	0.051	0.947	16.419	Sum In Feature 9	5.13	ECL	17:1 iso w9c
9.967	52286	0.057	0.947	16.475	Sum In Feature 4	2.98	ECL	17:1 iso I/anteiso B
10.092	17206	0.053	----	16.561		----		
10.193	111102	0.045	0.946	16.630	17:0 iso	6.32	ECL	Reference 0.000
10.329	99328	0.043	0.946	16.724	17:0 anteiso	5.65	ECL	Reference 0.001
10.376	45257	0.037	----	16.757		----		
10.426	52861	0.046	0.946	16.790	17:1 w8c	3.01	ECL	
10.529	111703	0.053	0.946	16.862	17:1 w6c	6.36	ECL	
10.665	110697	0.054	----	16.955		----		
10.728	57073	0.043	0.946	16.998	17:0	3.25	ECL	Reference -0.002
10.807	108400	0.060	0.946	17.051	16:1 2OH	6.17	ECL	
10.889	41634	0.038	----	17.107		----		
10.943	46414	0.050	0.946	17.144	16:0 iso 3OH	2.64	ECL	
11.028	80436	0.079	----	17.201		----		
11.173	37415	0.094	----	17.299		----	> max	
11.333	24737	0.070	0.946	17.408	17:0 10-methyl	1.41	ECL	
11.423	30754	0.112	0.947	17.468	18:1 iso H	----	> max	
11.664	23913	0.059	0.947	17.631	18:0 iso	1.36	ECL	Reference -0.002
11.792	18123	0.047	0.947	17.717	Sum In Feature 5	1.03	ECL	18:2 w6,9c/18:0 ante
11.867	24874	0.048	0.948	17.768	18:1 w9c	1.42	ECL	
11.943	9008	0.049	0.948	17.819	Sum In Feature 8	0.51	ECL	18:1 w7c
12.022	10683	0.068	----	17.873		----		
12.166	47180	0.069	----	17.971		----		
12.443	12996	0.136	0.950	18.159	17:0 iso 3OH	----	> max	
12.783	2037	0.061	0.951	18.390	18:0 10-methyl,	0.12	ECL	
12.992	711	0.045	0.953	18.532	17:0 3OH	0.04	ECL	
13.290	1343	0.044	0.954	18.735	19:0 anteiso	0.08	ECL	Reference 0.001
13.531	1079	0.044	0.956	18.899	19:0 cyclo w8c	0.06	ECL	
13.680	865	0.037	0.957	19.001	19:0	0.05	ECL	Reference -0.003
13.922	429	0.039	----	19.167		----		
14.597	1273	0.055	0.965	19.632	20:0 iso	0.07	ECL	Reference -0.008
15.437	510	0.037	----	20.210		----	> max rt	
15.624	222501	0.046	----	20.339		----	> max rt	
15.922	1494	0.052	----	20.544		----	> max rt	

16.152	42191	0.048	----	20.702		----	> max rt	
16.565	469	0.037	----	20.987		----	> max rt	
16.778	6667	0.056	----	21.134		----	> max rt	
16.880	1607	0.055	----	21.204		----	> max rt	
17.073	105380	0.047	----	21.337		----	> max rt	
17.219	121436	0.049	----	21.438		----	> max rt	
17.455	2006	0.066	----	21.600		----	> max rt	
17.585	11782	0.046	----	21.690		----	> max rt	
17.685	1909	0.046	----	21.759		----	> max rt	
18.413	2160	0.040	----	22.260		----	> max rt	
----	135281	---	----	----	Summed Feature 3	7.73	16:1	16:1 w6c/16:1 w7c
----	52286	---	----	----	Summed Feature 4	2.98	17:1 iso	17:1 anteiso B/iso I
----	18123	---	----	----	Summed Feature 5	1.03	18:2	18:0 ante/18:2 w6,9c
----	9008	---	----	----	Summed Feature 8	0.51	18:1 w7c	18:1 w6c
----	90050	---	----	----	Summed Feature 9	5.13	17:1 iso	16:0 10-methyl

S. saharensis DSM 45456^{T}								
RT	Response	Ar/Ht	RFact	ECL	Peak Name	Percent	Comment1	Comment2
0.038	926	0.040	----	3.124		----	< min rt	
0.313	2544	0.067	----	3.760		----	< min rt	
0.528	3216	0.087	----	4.257		----	< min rt	
0.707	2359	0.079	----	4.670		----	< min rt	
0.849	2215	0.079	----	4.998		----	< min rt	
1.103	2094	0.064	----	5.585		----	< min rt	
1.459	769	0.037	----	6.407		----	< min rt	
1.572	1734	0.057	----	6.670		----	< min rt	
1.710	5.07E+8	0.026	----	6.988	SOLVENT	----	< min rt	
1.808	6024	0.021	----	7.215		----	< min rt	
1.994	1423	0.034	----	7.650		----	< min rt	
2.078	1234	0.041	----	7.842		----	< min rt	
2.151	815	0.025	----	8.012		----	< min rt	
2.240	709	0.039	----	8.218		----	< min rt	
2.432	2140	0.065	----	8.660		----	< min rt	
2.498	5344	0.029	----	8.812		----	< min rt	
2.723	2889	0.045	----	9.331		----		
2.842	2278	0.042	1.149	9.606	10:0 iso	0.30	ECL deviates	Reference
3.007	1650	0.057	1.120	9.987	10:0	0.21	ECL deviates -	Reference -0.010
3.092	1724	0.057	----	10.134		----		
3.277	1413	0.067	----	10.445		----		
3.372	1017	0.035	1.082	10.605	11:0 iso	0.13	ECL deviates -	Reference
3.431	868	0.063	1.076	10.703	11:0 anteiso	0.11	ECL deviates	Reference
3.539	965	0.044	----	10.886		----		
3.613	917	0.039	1.059	11.007	11:0	0.11	ECL deviates	Reference
3.792	1626	0.054	----	11.235		----		
3.879	908	0.041	----	11.346		----		
4.087	915	0.037	1.029	11.612	12:0 iso	0.11	ECL deviates	Reference
4.125	972	0.036	----	11.661		----		
4.215	797	0.031	----	11.775		----		
4.281	497	0.034	----	11.860		----		
4.368	1497	0.039	----	11.971		----		
4.516	864	0.048	----	12.128		----		
4.596	597	0.032	----	12.209		----		
4.990	2818	0.030	0.990	12.613	13:0 iso	0.32	ECL deviates -	Reference
5.077	1297	0.030	0.987	12.702	13:0 anteiso	0.15	ECL deviates	Reference
5.364	1466	0.031	0.979	12.997	13:0	0.16	ECL deviates -	Reference -0.001
5.804	764	0.041	0.969	13.377	14:1 iso E	0.08	ECL deviates -	
5.877	917	0.036	----	13.441		----		
6.082	34571	0.033	0.963	13.618	14:0 iso	3.80	ECL deviates -	Reference
6.182	433	0.033	0.961	13.705	14:0 anteiso	0.05	ECL deviates -	Reference
6.521	6663	0.033	0.956	13.998	14:0	0.73	ECL deviates -	Reference
6.827	1257	0.054	----	14.234		----		
7.095	27546	0.037	0.949	14.440	15:1 iso G	2.99	ECL deviates	
7.207	9875	0.038	0.948	14.526	15:1 anteiso A	1.07	ECL deviates -	
7.335	171875	0.035	0.947	14.625	15:0 iso	18.59	ECL deviates	Reference
7.450	88585	0.034	0.946	14.714	15:0 anteiso	9.57	ECL deviates	Reference
7.631	12530	0.045	0.945	14.853	15:1 w6c	1.35	ECL deviates -	
7.820	21347	0.037	0.943	14.999	15:0	----	ECL deviates -	

8.060	1583	0.088	----	15.170		----	> max ar/ht	
8.186	976	0.049	----	15.260		----		
8.447	65531	0.051	0.941	15.447	16:1 iso G	7.04	ECL deviates	
8.704	211205	0.036	0.940	15.630	16:0 iso	22.68	ECL deviates	Reference
8.826	3154	0.038	0.940	15.717	16:0 anteiso	0.34	ECL deviates -	
8.965	11979	0.036	0.940	15.816	Sum In	1.29	ECL deviates -	16:1 w7c/16:1
8.999	11555	0.035	0.940	15.841	Sum In	1.24	ECL deviates -	16:1 w6c/16:1
9.133	3652	0.041	----	15.937		----		
9.218	23937	0.039	0.940	15.997	16:0	2.57	ECL deviates -	Reference -0.002
9.297	7396	0.039	----	16.051		----		
9.419	996	0.051	0.940	16.136	15:0 iso 3OH	0.11	ECL deviates	
9.539	2094	0.044	0.940	16.218	15:0 2OH	0.22	ECL deviates -	
9.627	904	0.045	----	16.279		----		
9.832	30673	0.054	0.940	16.420	Sum In	3.29	ECL deviates	17:1 iso w9c
9.997	11149	0.046	0.941	16.534	17:1 anteiso A	1.20	ECL deviates -	
10.137	20094	0.039	0.941	16.630	17:0 iso	2.16	ECL deviates	Reference -0.001
10.273	51688	0.041	0.942	16.724	17:0 anteiso	5.56	ECL deviates	Reference
10.372	27676	0.040	0.942	16.791	17:1 w8c	2.98	ECL deviates -	
10.472	47469	0.041	0.942	16.860	17:1 w6c	5.11	ECL deviates	
10.601	1330	0.041	----	16.950		----		
10.674	21608	0.044	0.943	17.000	17:0	2.33	ECL deviates	Reference -0.001
10.873	3711	0.056	----	17.135		----		
11.001	2201	0.073	----	17.221		----		
11.282	7990	0.038	0.946	17.412	17:0 10-methyl	0.86	ECL deviates	
11.389	1196	0.054	----	17.484		----		
11.538	909	0.048	0.948	17.585	18:3 w6c	0.10	ECL deviates	
11.608	2241	0.044	0.949	17.632	18:0 iso	0.24	ECL deviates	Reference -0.002
11.738	1695	0.042	0.950	17.721	Sum In	0.18	ECL deviates	18:2 w6,9c/18:0
11.811	4090	0.051	0.950	17.770	18:1 w9c	0.44	ECL deviates	
11.970	1875	0.043	----	17.878		----		
12.109	20423	0.049	----	17.972		----		
12.518	580	0.041	0.956	18.251	17:0 2OH	0.06	ECL deviates -	
12.925	1049	0.053	0.960	18.529	17:0 3OH	0.12	ECL deviates -	
13.253	319	0.031	0.963	18.752	Sum In	0.04	ECL deviates -	19:1 w11c/19:1
----	23534	---	----	----	Summed	2.53	16:1 w7c/16:1	16:1 w6c/16:1
----	1695	---	----	----	Summed	0.18	18:2	18:0 ante/18:2
----	319	---	----	----	Summed	0.04	19:1	19:1 w9c/19:1
----	30673	---	----	----	Summed	3.29	17:1 iso w9c	16:0 10-methyl

S. yanglingensis Hhs.015^{T}

RT	Response	Ar/Ht	RFact	ECL	Peak Name	Percent	Comment1	Comment2
0.066	2270	0.060	----	3.194		----	< min rt	
0.342	2487	0.077	----	3.831		----	< min rt	
0.494	3381	0.111	----	4.182		----	< min rt	
1.132	1941	0.058	----	5.655		----	< min rt	
1.248	432	0.039	----	5.923		----	< min rt	
1.483	582	0.034	----	6.467		----	< min rt	
1.597	939	0.050	----	6.729		----	< min rt	
1.710	5.064E+8	0.026	----	6.990	SOLVENT	----	< min rt	
1.809	3644	0.023	----	7.219		----	< min rt	
2.071	307	0.022	----	7.811		----	< min rt	
2.148	793	0.021	----	7.991		----	< min rt	
2.242	870	0.036	----	8.207		----	< min rt	
2.436	730	0.036	----	8.659		----	< min rt	
2.498	713	0.030	----	8.802		----	< min rt	
2.542	1006	0.046	----	8.904		----	< min rt	
2.726	3078	0.056	----	9.332		----		
2.843	2594	0.039	1.147	9.602	10:0 iso	0.51	ECL deviates -	Reference
3.015	1784	0.052	1.118	9.999	10:0	0.34	ECL deviates -	Reference
3.055	841	0.028	----	10.066		----		
3.106	1328	0.040	----	10.153		----		
3.234	833	0.039	----	10.369		----		
3.296	771	0.040	----	10.473		----		
3.378	1176	0.051	1.078	10.612	11:0 iso	0.22	ECL deviates	Reference
3.561	416	0.030	1.061	10.920	Sum In	0.08	ECL deviates	12:0 aldehyde ?
3.806	1868	0.066	----	11.253		----		
3.899	2172	0.065	----	11.372		----		
4.085	1629	0.035	1.026	11.610	12:0 iso	0.29	ECL deviates	Reference
4.134	1267	0.042	----	11.673		----		
4.215	1918	0.050	----	11.776		----		
4.379	1469	0.065	1.009	11.987	12:0	0.25	ECL deviates -	Reference -
4.524	1329	0.054	----	12.138		----		
4.596	828	0.036	----	12.212		----		
4.990	2067	0.033	0.987	12.616	13:0 iso	0.35	ECL deviates	Reference
5.081	773	0.034	0.984	12.709	13:0 anteiso	0.13	ECL deviates	Reference
5.234	1038	0.059	----	12.866		----		
5.362	212	0.020	0.975	12.997	13:0	0.04	ECL deviates -	Reference -
5.879	2059	0.045	0.964	13.445	12:0 3OH	0.34	ECL deviates -	
6.082	51914	0.032	0.961	13.622	14:0 iso	8.55	ECL deviates	Reference
6.400	1938	0.092	0.955	13.896	14:1 w5c	----	> max ar/ht	
6.520	1759	0.042	0.954	14.000	14:0	0.29	ECL deviates	Reference -
6.828	1149	0.060	----	14.238		----		
7.094	19928	0.037	0.947	14.442	15:1 iso G	3.24	ECL deviates	
7.206	2773	0.041	0.946	14.529	15:1 anteiso A	0.45	ECL deviates	
7.333	52726	0.034	0.945	14.626	15:0 iso	8.55	ECL deviates	Reference
7.447	18534	0.035	0.944	14.714	15:0 anteiso	3.00	ECL deviates	Reference -
7.631	6530	0.045	0.943	14.855	15:1 w6c	1.06	ECL deviates -	
7.820	2003	0.037	0.942	15.001	15:0	----	ECL deviates	
8.190	1836	0.064	----	15.264		----		
8.459	77551	0.048	0.940	15.456	16:1 iso H	12.50	ECL deviates -	

8.703	128497	0.035	0.940	15.630	16:0 iso	20.71	ECL deviates	Reference
8.815	3434	0.049	0.939	15.709	16:0 anteiso	0.55	ECL deviates -	
9.001	38604	0.055	0.939	15.842	Sum In	6.22	ECL deviates -	16:1 w6c/16:1
9.133	3106	0.042	----	15.936		----		
9.219	7107	0.038	0.940	15.998	16:0	1.15	ECL deviates -	Reference -
9.297	6089	0.037	----	16.051		----		
9.539	1068	0.046	0.940	16.217	15:0 2OH	0.17	ECL deviates -	
9.632	436	0.033	----	16.281		----		
9.830	26752	0.045	0.941	16.417	Sum In	4.32	ECL deviates	17:1 iso w9c
9.987	5546	0.042	0.941	16.525	17:1 anteiso	0.90	ECL deviates	
10.139	5165	0.037	0.942	16.629	17:0 iso	0.83	ECL deviates -	Reference
10.272	14384	0.041	0.942	16.721	17:0 anteiso	2.33	ECL deviates -	Reference -
10.373	20829	0.040	0.943	16.790	17:1 w8c	3.37	ECL deviates -	
10.475	80176	0.041	0.943	16.860	17:1 w6c	12.98	ECL deviates	
10.602	1479	0.046	----	16.948		----		
10.675	3412	0.049	0.945	16.998	17:0	0.55	ECL deviates -	Reference -
10.847	1332	0.061	----	17.113		----		
11.011	1541	0.069	0.947	17.224	16:0 2OH	0.25	ECL deviates -	
11.279	8053	0.040	0.948	17.405	17:0 10-	1.31	ECL deviates -	
11.369	2005	0.052	0.949	17.466	18:1 iso H	0.33	ECL deviates	
11.612	2235	0.045	0.951	17.629	18:0 iso	0.36	ECL deviates -	Reference
11.736	6604	0.044	0.952	17.713	Sum In	1.08	ECL deviates -	18:2 w6,9c/18:0
11.810	9966	0.043	0.953	17.763	18:1 w9c	1.63	ECL deviates -	
11.886	865	0.036	0.953	17.814	Sum In	0.14	ECL deviates -	18:1 w7c
11.967	1788	0.041	----	17.869		----		
12.109	8267	0.050	----	17.964		----		
12.310	601	0.040	----	18.100		----		
12.392	2901	0.043	0.958	18.156	17:0 iso 3OH	0.48	ECL deviates -	
13.642	936	0.061	0.971	19.004	19:0	0.16	ECL deviates	Reference
17.841	2262	0.051	----	21.902		----	> max rt	
18.488	6091	0.082	----	22.347		----	> max rt	
----	416	---	----	----	Summed	0.08	12:0	unknown
----	-----	---	----	----		----	16:1 iso I/14:0	14:0 3OH/16:1
----	38604	---	----	----	Summed	6.22	16:1 w7c/16:1	16:1 w6c/16:1
----	6604	---	----	----	Summed	1.08	18:2	18:0 ante/18:2
----	865	---	----	----	Summed	0.14	18:1 w7c	18:1 w6c
----	26752	---	----	----	Summed	4.32	17:1 iso w9c	16:0 10-methyl

S. longispora JCM 3314^{T}								
RT	Response	Ar/Ht	RFact	ECL	Peak Name	Percent	Comment1	Comment2
0.036	369	0.026	----	3.123		----	< min rt	
0.308	2466	0.071	----	3.750		----	< min rt	
0.455	2767	0.093	----	4.091		----	< min rt	
0.704	2126	0.080	----	4.665		----	< min rt	
0.844	1637	0.065	----	4.989		----	< min rt	
1.105	2114	0.059	----	5.590		----	< min rt	
1.572	794	0.041	----	6.670		----	< min rt	
1.709	4.975E+8	0.027	----	6.987	SOLVENT	----	< min rt	
1.873	2133	0.023	----	7.366		----	< min rt	
2.073	2649	0.025	----	7.829		----	< min rt	
2.148	612	0.022	----	8.001		----	< min rt	
2.195	467	0.059	----	8.111		----	< min rt	
2.277	437	0.024	----	8.300		----	< min rt	
2.429	598	0.043	----	8.650		----	< min rt	
2.498	1894	0.025	----	8.811		----	< min rt	
2.720	2503	0.057	----	9.323		----		
2.843	2341	0.043	1.147	9.606	10:0 iso	0.40	ECL deviates	Reference
3.014	2115	0.054	1.118	10.001	10:0	0.35	ECL deviates	Reference
3.089	1652	0.064	----	10.127		----		
3.275	1569	0.073	----	10.440		----		
3.367	2127	0.080	1.078	10.595	11:0 iso	0.34	ECL deviates -	Reference -
3.541	1668	0.060	----	10.888		----		
3.610	1129	0.037	1.056	11.003	11:0	0.18	ECL deviates	Reference
3.783	1652	0.063	----	11.223		----		
3.887	1062	0.054	----	11.357		----		
4.087	1483	0.046	1.026	11.612	12:0 iso	0.23	ECL deviates	Reference
4.214	865	0.032	----	11.775		----		
4.279	495	0.038	----	11.858		----		
4.512	846	0.044	----	12.125		----		
4.594	209	0.017	----	12.208		----		
4.990	1666	0.032	0.987	12.614	13:0 iso	0.25	ECL deviates	Reference
5.079	573	0.036	0.984	12.705	13:0 anteiso	0.08	ECL deviates	Reference
5.364	243	0.026	0.975	12.998	13:0	0.04	ECL deviates -	Reference -
5.881	1298	0.035	0.964	13.445	12:0 3OH	0.19	ECL deviates -	
6.083	30003	0.033	0.961	13.620	14:0 iso	4.31	ECL deviates	Reference
6.437	11888	0.039	----	13.927		----		
6.519	3455	0.047	0.954	13.998	14:0	0.49	ECL deviates -	Reference -
7.095	21204	0.037	0.947	14.441	15:1 iso G	3.01	ECL deviates	
7.206	3110	0.041	0.946	14.527	15:1 anteiso A	0.44	ECL deviates	
7.332	45159	0.033	0.945	14.624	15:0 iso	6.39	ECL deviates	Reference
7.450	17442	0.034	0.944	14.714	15:0 anteiso	2.47	ECL deviates	Reference
7.557	3413	0.033	0.944	14.797	15:1 w8c	0.48	ECL deviates	
7.630	8892	0.048	0.943	14.853	15:1 w6c	1.26	ECL deviates -	
7.732	35584	0.042	----	14.932		----		
7.853	32443	0.050	0.942	15.023	15:0	----	ECL deviates	
8.190	291	0.025	----	15.264		----		
8.257	1402	0.042	----	15.312		----		
8.458	55013	0.050	0.940	15.454	16:1 iso H	7.74	ECL deviates -	
8.701	96362	0.037	0.940	15.628	16:0 iso	13.55	ECL deviates	Reference

8.810	5012	0.045	----	15.706		----		
9.001	73915	0.073	0.939	15.842	Sum In	10.40	ECL deviates -	16:1 w6c/16:1
9.139	121874	0.041	----	15.941		----		
9.218	21931	0.046	0.940	15.997	16:0	3.08	ECL deviates -	Reference -
9.295	23594	0.070	----	16.050		----		
9.438	14174	0.067	----	16.149		----		
9.537	12734	0.072	0.940	16.217	15:0 2OH	1.79	ECL deviates -	
9.669	22425	0.075	----	16.308		----		
9.831	47158	0.061	0.941	16.419	Sum In	6.64	ECL deviates	17:1 iso w9c
9.988	16390	0.070	0.941	16.528	17:1 anteiso	2.31	ECL deviates	
10.138	12836	0.051	0.942	16.630	17:0 iso	1.81	ECL deviates	Reference -
10.272	26452	0.049	0.942	16.723	17:0 anteiso	3.73	ECL deviates	Reference -
10.373	44168	0.049	0.943	16.792	17:1 w8c	6.23	ECL deviates	
10.476	77836	0.041	0.943	16.863	17:1 w6c	10.99	ECL deviates	
10.613	12177	0.060	----	16.957		----		
10.673	12333	0.047	0.945	16.999	17:0	1.74	ECL deviates -	Reference -
10.736	25423	0.067	0.945	17.041	16:1 2OH	3.60	ECL deviates -	
10.865	23459	0.088	----	17.128		----	> max ar/ht	
11.004	12629	0.084	----	17.223		----	> max ar/ht	
11.173	6676	0.065	----	17.337		----		
11.279	13154	0.047	0.948	17.409	17:0 10-	1.87	ECL deviates	
11.368	9691	0.109	0.949	17.469	18:1 iso H	----	> max ar/ht	
11.612	6416	0.066	0.951	17.634	18:0 iso	0.91	ECL deviates	Reference
11.737	3829	0.050	0.952	17.718	Sum In	0.55	ECL deviates -	18:2 w6,9c/18:0
11.810	8976	0.048	0.953	17.768	18:1 w9c	1.28	ECL deviates -	
11.883	2486	0.046	0.953	17.817	Sum In	0.35	ECL deviates -	18:1 w7c
11.967	4667	0.052	----	17.874		----		
12.112	14333	0.055	----	17.972		----		
12.391	3518	0.056	0.958	18.162	17:0 iso 3OH	0.50	ECL deviates	
18.358	11752	0.130	----	22.261		----	> max rt	
----	73915	---	----	----	Summed	10.40	16:1 w7c/16:1	16:1 w6c/16:1
----	3829	---	----	----	Summed	0.55	18:2	18:0 ante/18:2
----	2486	---	----	----	Summed	0.35	18:1 w7c	18:1 w6c
----	47158	---	----	----	Summed	6.64	17:1 iso w9c	16:0 10-methyl

Table S4. The fatty acid composition of strain HUAS TT1^T, *S. hoggarensis* DSM 45457^T, *S. saharensis* DSM 45456^T, *S. yanglingensis* CGMCC 4.5627^T and *S. longispora* CGMCC 4.1357^T.

Fatty acid (%)	1	2	3	4	5
iso-$C_{14:0}$	1.9	2.3	3.8	8.6	4.3
iso-$C_{15:0}$	11.0	4.7	18.6	8.6	6.4
anteiso-$C_{15:0}$	1.8	19.1	9.6	3.0	2.5
iso-$C_{15:1}$ G	–	Tr	3.0	3.2	3.0
$C_{16:0}$	–	–	2.6	1.2	3.1
$C_{16:1}$ 2-OH	0.8	6.2	–	–	3.6
anteiso-$C_{16:0}$	0.6	0.7	Tr	0.6	–
iso-$C_{16:0}$	14.7	13	22.7	20.7	13.6
iso-$C_{16:0}$ H	–	–	–	12.5	–
iso-$C_{16:1}$ H	–	2.1	–	–	7.8
iso-$C_{16:1}$ G	2.0	–	7.0	–	–
iso-$C_{16:0}$ 3-OH	–	2.6	–	–	–
$C_{17:0}$	1.1	3.3	2.3	0.6	1.7
anteiso-$C_{17:0}$	2.3	5.7	5.6	2.3	3.7
iso-$C_{17:0}$	1.8	6.0	2.2	0.8	1.8
10-methy1 $C_{17:0}$	Tr	1.4	0.9	1.3	1.9
$C_{17:1}$ ω6c	5.3	6.4	5.1	13.0	11.0
$C_{17:1}$ ω8c	1.4	3.0	3.0	3.4	6.2
anteiso-$C_{17:1}$ ω9c	Tr	–	–	0.9	2.3
anteiso-$C_{17:1}$ A	–	–	1.2	–	–
$C_{18:0}$	3.2	–	–	–	–
iso-$C_{18:0}$	Tr	1.4	Tr	Tr	0.9
$C_{18:1}$ ω9c	14.0	1.4	Tr	1.6	1.3
Summed Feature 3	3.0	7.7	2.5	6.2	10.4
Summed Feature 5	14.6	1.0	Tr	1.1	0.6
Summed Feature 9	4.0	5.1	3.3	4.3	6.6

Note: 1, HUAS TT1^T; 2, *S. hoggarensis* DSM 45457^T; 3, *S. saharensis* DSM 45456^T; 4, *S. yanglingensis* CGMCC 4.5627^T; 5, *S. longispora* CGMCC 4.1357^T. Tr, trace amount (<0.5%); –, not detected; Summed Feature 3, $C_{16:1}$ ω7c/$C_{16:1}$ ω9c; Summed Feature 5, $C_{18:2}$ ω6,9c /$C_{18:0}$ ante; Summed Feature 9, *iso*-$C_{17:1}$ ω9c. All data were from this study.

Table S5. Genome features of strains HUAS TT1^{T}, *S. hoggarensis* CCUG 60214^{T}, *S. saharensis* DSM 45456^{T}, *S. yanglingensis* Hhs.015^{T} and *S. longispora* JCM 3314^{T}.

Strains	1	2	3	4	5
Size (bp)	8,515,408	7,622,816	8,924,967	8,286,303	8,393,791
Genes (total)	7,761	7,408	8,073	7,400	7,460
CDSs (total)	7,674	7,352	8,006	7,338	7,391
Genes (coding)	7,581	7,066	7,921	7,209	7,292
CDSs (with protein)	7,581	7,066	7,921	7,209	7,292
Genes (RNA)	87	56	67	62	69
rRNAs	12	3	12	5	12
complete rRNAs	12	3	12	5	12
tRNAs	61	50	52	54	54
ncRNAs	14	3	3	3	3
Pseudo Genes (total)	93	286	85	129	99
CDSs (without protein)	93	286	85	129	99
Pseudo Genes (ambiguous residues)	0	0	0	0	0
Pseudo Genes (frameshifted)	24	16	22	47	39
Pseudo Genes (incomplete)	77	274	74	104	69
Pseudo Genes (internal stop)	5	3	5	2	1
Pseudo Genes (multiple problems)	11	7	15	24	10
DNA G+C content	72.7	72.3	72.4	73.4	73.0
Accession numbers	CP160453.1	GCA_042678885.1	GCA_006716745.1	GCA_030852425.1	GCA_039535375.1

Note: 1, HUAS TT1^{T}; 2, *S. hoggarensis* CCUG 60214^{T}; 3, *S. saharensis* DSM 45456^{T}; 4, *S. yanglingensis* Hhs.015^{T}; 5, *S. longispora* JCM 3314^{T}.

Table S6. The subsystem category number of genes of strains HUAS TT1[T], *S. hoggarensis* CCUG 60214[T], *S. saharensis* DSM 45456[T], *S. yanglingensis* Hhs.015[T] and *S. longispora* JCM 3314[T] based on RAST annotation server.

Subsystem Feature Counts	1	2	3	4	5
Amino Acids and Derivatives	392	373	394	418	422
Carbohydrates	319	298	306	278	308
Protein Metabolism	203	204	216	206	193
Cofactors, Vitamins, Prosthetic Groups, Pigments	175	187	183	173	178
Fatty Acids, Lipids, and Isoprenoids	164	167	172	160	160
Nucleosides and Nucleotides	104	103	103	104	105
DNA Metabolism	103	110	106	104	100
Respiration	93	95	93	87	93
RNA Metabolism	50	52	49	50	48
Stress Response	44	38	47	48	47
Metabolism of Aromatic Compounds	43	26	45	36	36
Cell Wall and Capsule	39	45	45	42	40
Virulence, Disease and Defense	37	47	47	44	40
Miscellaneous	34	38	34	35	35
Iron acquisition and metabolism	31	33	3	37	36
Phosphorus Metabolism	29	29	28	25	26
Membrane Transport	27	24	30	26	29
Regulation and Cell signaling	22	17	19	18	22
Nitrogen Metabolism	18	15	34	15	26
Secondary Metabolism	13	8	12	13	15
Phages, Prophages, Transposable elements, Plasmids	7	0	4	0	0
Potassium metabolism	7	4	5	7	7
Sulfur Metabolism	7	9	15	9	9
Dormancy and Sporulation	1	1	3	1	1
Motility and Chemotaxis	0	0	0	1	1
Photosynthesis	0	0	0	4	0
Nodulation	0	0	0	0	0
Cell Division and Cell Cycle	0	0	0	0	0

Note: 1, HUAS TT1[T]; 2, *S. hoggarensis* CCUG 60214[T]; 3, *S. saharensis* DSM 45456[T]; 4, *S. yanglingensis* Hhs.015[T]; 5, *S. longispora* JCM 3314[T].

Table S7. The distribution of biosynthetic gene clusters in the genome of strains HUAS TT1^T, *S. hoggarensis* CCUG 60214^T, *S. saharensis* DSM 45456^T, *S. yanglingensis* Hhs.015^T and *S. longispora* JCM 3314^T by antiSMASH analyses.

	1	2	3	4	5
T1PKS	8	4	7	13	8
NRPS	8	14	6	11	9
Terpene	3	3	6	5	7
PKS-like	3	1	3	1	0
NRP-metallophore	2	1	1	1	1
Arylpolyene	1	2	0	0	0
Ectoine	1	2	1	0	0
HgIE-KS	1	2	0	3	2
Hydrogen-cyanide	1	0	0	0	0
Indole	1	1	1	1	2
LAP, NI-siderophore	1	1	1	1	1
Lanthipeptide-class-i	1	0	0	0	1
Lanthipeptide-class-ii	1	1	1	1	2
NAPAA	1	1	1	1	0
Ligosaccharide	1	0	0	0	0
Other	1	2	2	2	1
RRE-containing	1	0	1	0	2
Thiopeptide	1	0	1	0	1
Acyl-amino-acids	0	0	0	1	1
Betalactone	0	0	0	1	1
Butyrolactone	0	0	1	1	1
Hserlactone	0	0	0	0	1
Lanthipeptide-class-iii	0	0	1	1	2
Lassopeptide	0	1	0	0	1
NRPS-like	0	6	5	5	4
Nucleoside	0	0	0	0	1
RiPP-like	0	2	4	1	2
T2PKS	0	0	1	1	1
T3PKS	0	0	2	1	2
TransAT-PKS	0	1	0	1	1
HR-T2PKS	0	0	2	0	0
Oligosaccharide	0	0	1	0	0
Melanin	0	0	1	0	0
Prodigiosin	0	0	1	0	0
Redox-cofactor	0	0	1	0	0
Thioamide-NRP	0	0	1	0	0

Note: 1, HUAS TT1^T; 2, *S. hoggarensis* CCUG 60214^T; 3, *S. saharensis* DSM 45456^T;

4, *S. yanglingensis* Hhs.015^T; 5, *S. longispora* JCM 3314^T. T1PKS, Type I PKS (Polyketide synthase); NRPS, Non-ribosomal peptide synthetase; terpene; PKS-like, Other types of PKS; NRP-metallophore, Non-ribosomal peptide metallophores; arylpolyene, Aryl polyene; ectoine; hglE-KS, Heterocyst glycolipid synthase-like PKS; hydrogen-cyanide, Hydrogen cyanide (AF208523); indole; LAP, Linear azol(in)e-containing peptides; lanthipeptide-class-i, Class I lanthipeptides like nisin; lanthipeptide-class-ii, Class II lanthipeptides like mutacin II (U40620); NAPAA, Non-alpha poly-amino acids like e-Polylysin; NI-siderophore, NRPS-independent, IucA/IucC-like siderophores (siderophoren prior to 7.0); oligosaccharide, RRE-containing, RRE-element containing cluster; thiopeptide contaimning cluster; acyl-amino-acids, N-acyl amino acid; betalactone, Beta-lactone containing protease inhibitor; butyrolactone, Butyrolactone; hserlactone, Homoserine lactone; lanthipeptide-class-iii, Class III lanthipeptides like labyrinthopeptin (FN178622); lassopeptide; NRPS-like, NRPS-like fragment; nucleoside; RiPP-like, Other unspecified ribosomally synthesised and post-translationally modified peptide product (RiPP); T2PKS, Type II PKS; T3PKS, Type III PKS; TransAT-PKS domain not found; other, Cluster containing a secondary metabolite-related protein that does not fit into any other category; HR-T2PKS, Highly reducing type II PKS like ishigamide and skyllamycin; Prodigiosin, Serratia-type non-traditional PKS prodigiosin biosynthesis pathway; Redox-cofactor, Redox-cofactors such as PQQ (NC_021985:1458906-1494876); Thioamide-NRP; Thioamide-containing non-ribosomal peptide.

Table S8. Genome relatedness between strain HUAS TT1^T and related reference strains.

	dDDH(%)											
ANIb/m(%)	1	2	3	4	5	6	7	8	9	10	11	12
1		86.3	23.5	20.2	20.2	19.8	20.1	20.9	34.60	25.90	27.70	24.60
2	98.2/98.5		24.0	20.9	20.4	19.9	19.9	20.6	33.30	26.50	28.50	25.50
3	79.6/85.2	80.1/85.6		20.9	19.9	19.3	20.4	21.0	38.10	25.70	27.50	24.60
4	75.9/83.3	76.6/84.3	76.4/84.0		20.3	20.7	20.9	19.8	27.50	25.10	27.40	24.30
5	69.2/85.0	69.3/85.1	69.3/85.1	69.2/85.8		20.0	19.1	19.9	27.70	25.20	27.60	24.50
6	69.5/85.5	69.5/85.1	69.5/84.9	70.0/85.6	72.7/84.4		18.8	19.5	27.80	25.20	32.80	24.60
7	69.6/85.0	69.5/84.8	69.6/85.25	69.8/85.1	70.1/85.0	76.6/84.1		19.8	23.30	23.40	23.00	22.90
8	69.3/85.8	69.4/85.2	69.6/85.9	69.5/84.9	72.0/85.2	72.3/84.6	70.2/84.3		24.60	27.00	24.60	25.70
9	86.44/89.53	86.00/88.80	88.31/90.50	82.61/86.85	82.72/86.88	82.48/86.87	78.29/85.03	79.66/85.64		25.80	27.50	24.40
10	80.29/86.00	81.04/86.12	80.36/85.91	79.79/85.77	79.82/85.75	79.81/85.79	78.39/85.00	81.45/86.35	80.26/85.90		25.40	27.60
11	82.49/87,01	83.01/86.83	82.18/86.76	82.69/86.95	82.75/86.91	85.13/89.10	77.86/85.13	79.27/85.77	82.34/86.70	79.82/85.82		24.40
12	79.42/85,59	79.90/85.74	79.32/85.52	78.94/85.46	78.89/85.43	78.79/85.53	78.07/84.93	80.12/85.91	79.38/85.45	81.86/86.44	79.01/85.57	
13	85.99/89.44	85.99/88.82	88.04/90.63	82.16/86.75	82.01/86.69	81.57/86.78	78.06/85.01	79.46/85.58	90.07/92.46	80.03/85.82	81.83/86.59	78.90/85.39
14	83.04/87.25	82.85/86.85	82.61/86.80	83.14/87.21	83.25/87.22	89.73/91.73	77.89/85.11	79.46/85.57	83.01/85.78	79.77/85.74	85.97/89.03	78.94/85.51
15	79.65/85.99	80.45/85.95	79.75/85.83	79.52/85.67	79.56/85.71	79.08/85.63	78.52/85.02	80.67/86.19	79.78/85.83	84.10/87.42	79.12/85.71	82.46/86.61
16	84.14/88.21	86.07/88.68	84.50/88.06	81.37/86.28	81.49/86.31	80.93/86.32	77.52/84.91	78.78/85.35	84.71/88.35	79.80/85.71	80.63/86.20	78.46/85.25
17	85.01/88.98	86.35/89.22	85.24/88.79	82.05/86.65	81.99/86.64	81.22/86.70	78.08/85.02	79.69/85.63	85.31/88.95	80.39/86.12	81.11/86.57	79.08/85.47
18	80.41/86.02	80.26/85.88	80.18/85.82	79.89/85.78	79.96/85.80	80.26/86.02	77.73/85.08	79.21/85.70	80.01/85.85	79.48/85.88	80.97/86.40	78.69/85.56
19	86.26/89.46	86.37/88.91	88.20/90.78	82.33/86.79	82.40/86.78	82.05/86.85	78.17/85.13	79.59/85.71	88.09/90.65	80.28/85.90	82.13/86.67	79.09/85.52
20	80.64/86.01	80.88/86.18	80.57/85.98	80.08/85.86	79.95/85.87	80.17/85.93	78.42/85.06	81.42/86.34	80.52/85.94	84.42/88.20	80.32/85.99	82.42/86.76
21	81.74/86.29	80.81/86.25	81.64/86.08	81.47/86.02/	81.49/86.04	81.34/86.11	78.71/85.24	82.49/86.51	81.50/86.07	86.14/88.32	81.47/86.20	83.34/86.70
22	77.28/85.14	77.64/85.21	76.92/84.99	77.31/85.11	77.41/85.09	78.00/85.03	76.07/84.92	71.00/85.00	77.81/85.01	76.49/85.05	78.28/85.15	76.42/84.98
23	77.35/85.16	77.66/85.21	77.02/85.07	77.53/85.20	77.49/85.22	78.10/85.13	70.10/84.94	77.27/85.07	77.71/85.02	76.53/85.05	78.18/85.20	76.53/84.93
24	76.81/84.51	77.21/84.69	76.65/8.58	76.73/84.56	76.79/84.51	75.28/84.39	75.54/84.41	75.97/84.48	76.73/85.57	76.24/84.58	75.49/84.49	75.85/84.44

Table S8. (continued).

	dDDH(%)											
ANIb/m(%)	13	14	15	16	17	18	19	20	21	22	23	24
1	34.40	28.70	25.30	31.60	33.30	25.30	34.70	25.50	26.50	22.70	22.90	21.50
2	33.40	28.70	26.00	33.40	34.70	25.60	33.90	26.70	26.70	23.50	23.50	22.30
3	38.80	27.90	25.10	31.50	33.00	25.30	39.20	25.60	26.20	22.80	23.00	21.70
4	27.50	28.60	24.90	26.90	27.50	24.90	27.70	25.20	25.90	22.60	22.80	21.30
5	27.40	28.80	24.90	27.00	27.60	24.90	27.70	25.40	26.10	22.70	22.90	21.50
6	27.60	41.30	24.80	27.10	27.60	25.50	27.80	25.50	26.10	23.00	23.10	21.50
7	22.90	23.20	23.40	22.80	23.10	23.10	23.30	23.60	24.10	22.20	22.40	20.90
8	24.50	24.40	26.40	24.20	24.70	24.60	24.90	26.90	27.40	22.70	22.80	21.40
9	44.80	27.90	25.20	32.20	33.40	25.10	38.80	25.60	26.20	22.60	22.70	21.50
10	25.50	25.20	29.80	25.40	26.10	25.30	25.60	32.10	32.20	23.20	23.20	21.90
11	27.10	32.70	24.70	26.70	27.30	26.00	27.40	25.40	26.30	23.00	23.10	21.50
12	24.30	24.60	28.00	24.10	24.40	24.50	24.60	28.10	28.30	22.70	22.80	21.60
13		27.50	24.80	32.50	34.20	24.80	40.10	25.40	25.80	22.70	22.50	21.50
14	82.19/86.64		24.80	26.70	27.60	25.50	27.80	25.30	26.30	22.90	23.00	21.50
15	79.13/85.70	79.72/85.74		24.70	24.90	24.90	25.00	28.90	29.50	23.00	23.10	21.70
16	84.32/88.45	81.52/86.14	79.26/85.77		35.20	24.40	32.40	25.40	25.50	22.50	22.50	21.60
17	85.29/89.18	82.09/86.55	79.14/85.81	85.65/89.49		24.70	34.10	25.70	26.00	22.60	22.70	21.80
18	79.58/85.67	80.27/86.05	79.14/85.81	79.19/85.36	79.59/85.70		25.00	25.80	26.10	24.10	24.20	22.30
19	88.48/91.02	81.80/86.68	79.64/85.74	84.97/88.45	85.46/89.15	79.84/85.74		25.90	26.10	22.80	22.80	21.70
20	80.20/85.84	82.95/87.05	82.95/87.05	79.71/85.59	80.30/85.94	79.76/85.94	80.50/85.96		33.10	23.10	23.10	21.70
21	81.21/85.95	84.16/87.22	84.16/87.22	80.75/85.68	81.44/86.04	81.23/86.24	81.57/86.02	86.31/88.77		23.60	23.60	22.20
22	77.31/84.97	77.35/85.06	77.35/85.06	76.88/84.81	77.30/85.04	78.20/85.56	76.57/85.05	77.27/85.07	78.48/85.29		61.90	21.20
23	77.24/84.94	78.05/85.05	77.30/85.17	76.99/84.91	77.39/84.99	78.20/85.49	76.60/85.01	77.29/85.23	78.60/85.41	95.18/95.85		21.30
24	76.02/84.53	76.81/84.43	76.10/84.54	75.85/84.42	75.33/84.59	77.26/84.73	76.32/84.52	76.72/84.54	77.54/84.65	76.08/84.39	76.02/84.40	

Note: ANI, average nucleotide identity; dDDH, digital DNA–DNA hybridization. The strains 1-24 are the same as those of Table S1.
