# Supplementary material for: Saccharothrix camelliae sp. nov., isolated from rhizosphere soil of Camellia oleifera Abel and proposal of Saccharothrixyanglingensis as a later heterotypic synonym of Saccharothrix longispora
Source: Front Microbiol. 2026 Feb 27;17:1716500. doi: 10.3389/fmicb.2026.1716500 (PMC12983401; doi:10.3389/fmicb.2026.1716500)
Supplement: Supplementary file 5 [file Data_Sheet_5.PDF]

The authors have addressed several earlier comments and improved the manuscript substantially, including adding polar lipid profiles, providing raw sequencing data accession numbers, incorporating EasyCGTree phylogenomics, and correcting many typographical issues. However, several core scientific and methodological requirements are still incomplete, and the manuscript remains below the full reproducibility and transparency standards expected by Frontiers in Microbiology and IJSEM. Therefore, the manuscript still requires Major Revision before it can be considered for acceptance.

Below are the specific remaining deficiencies that must be addressed:

1. Although the authors now provide an SRA accession number for raw genome reads, the manuscript still does not include essential genome assembly metadata. The authors must clearly state the sequencing platform used, total coverage depth, assembler name and version, assembly parameters, and whether the genome was circularized or left as a linear contig. This information must appear explicitly in the Methods section for full reproducibility.

Thank you for your suggestion. Added, please see line 128-129. The detailed methods were described according to Chen et. al (Chen et al., 2022). This article was published by our research group.

Chen, D., Mo, P., and Li, B.Y. (2022). Complete Genome Sequence of *Nocardiopsis exhalans* Strain JCM 11759^T, Isolated from Indoor Air of a Water-Damaged Private House in Finland. *Microbiol Resour Ann*. 11, e00930-22. https://doi.org/10.1128/mra.00930-22

2. The genome-based comparison results (ANI and dDDH) are presented, but the manuscript still lacks the full raw output files and detailed methodological parameters. The study must include:

– JSpecies parameters (fragment size, BLAST vs NUCmer settings, version used),
– GGDC formula applied (d4, d6, etc.) and confidence intervals,
– complete ANI and dDDH pairwise matrices as supplementary data,
– Newick tree files and alignment files for both the 16S rRNA gene tree and the genome-based phylogeny (TYGS and EasyCGTree).

These raw data files and parameters are mandatory for verification of taxonomic conclusions.

Thank you for your suggestion. Added.

3. The authors added MIDI fatty-acid data for the new strain but confirm that raw MIDI chromatograms and peak tables for all reference strains were lost. This is a significant problem. For chemotaxonomy to be valid, all strains must be grown under identical conditions and their raw chromatograms/peak tables must be provided. The authors must either:

– regenerate the fatty-acid profiles for all reference strains under the same growth conditions and provide the raw chromatograms and peak tables, or
– provide documented justification and reproducible replacement analyses.

Without complete chemotaxonomic raw data, the phenotypic delineation is insufficiently supported.

Thank you for your suggestion. Added, please see Table S2 and Figure S3. We did the experiment again.

4. The new EasyCGTree phylogeny added by the authors is appreciated and improves the genomic analysis. However, the manuscript still fails to document the exact parameters used in all phylogenetic analyses. The authors must state:

– alignment program and trimming strategy,

– substitution/evolutionary model used,
– phylogenetic inference algorithm,
– number of bootstrap replicates,
– EasyCGTree version and core-gene set used.

These details are required so that the phylogeny can be reproduced by other researchers.

Thank you for your suggestion. Added.

5. The proposed heterotypic synonymization of *S. yanglingensis* with *S. longispora* requires complete and transparent documentation. The comparative phenotypic and chemotaxonomic table should be clearly expanded to include all diagnostic characters side-by-side, and the emended description of S. longispora must fully incorporate these data. The genome accession numbers used for comparisons should be explicitly listed in the main text.

Thank you for your suggestion. Added.

6. Culture collection deposition certificates have now been provided and the type-strain designations seem correct; however, the manuscript should explicitly state the deposit numbers and their availability under the Data Availability section. This is required for valid species publication.

Thank you so much.

7. The authors state that speculative antiSMASH interpretations have been removed; however, all remaining statements about metabolic potential should be conservative. If antiSMASH summaries are retained, they should remain strictly descriptive and the raw antiSMASH output should be provided as supplementary material.

Thank you for your suggestion. Added.

8. Several technical details remain unclear or missing in the Methods section, including:
– explicit settings for all genome comparison tools,
– criteria for phenotype scoring (API, Biolog),
– detailed growth conditions for chemotaxonomic analyses,
– number of biological replicates used.

These should be clearly defined to ensure reproducibility.

Thank you for your suggestion. Added.

9. The revised manuscript is improved linguistically but still requires another careful proofreading round for grammar, consistency, and formatting. Some inconsistencies in strain numbering, citation formatting, and minor typographical issues remain and should be corrected before final acceptance.

Thank you for your suggestion. We have tried our best to modify it.

Although the manuscript is significantly improved, essential raw data and methodological details remain incomplete. To meet the standards of Frontiers in Microbiology and IJSEM for valid species description and nomenclatural actions, the authors must address all points above. Once the missing genome outputs, chemotaxonomic raw data, phylogenetic parameters, and assembly metadata are fully added and verified, the manuscript will be suitable for reconsideration. At this stage, a Major Revision is required.

Thank you for your suggestion. We have revised all the problems mentioned above.
