# Supplementary material for: Saccharothrix camelliae sp. nov., isolated from rhizosphere soil of Camellia oleifera Abel and proposal of Saccharothrixyanglingensis as a later heterotypic synonym of Saccharothrix longispora
Source: Front Microbiol. 2026 Feb 27;17:1716500. doi: 10.3389/fmicb.2026.1716500 (PMC12983401; doi:10.3389/fmicb.2026.1716500)
Supplement: Supplementary file 4 [file Data_Sheet_4.PDF]

A Cover Letter

Dear editor Prof. Govindan Rajamohan, editor Prof. Peter Mullany, reviews and Frontiers in Microbiology team,

Thank you for sending me the comments. Taking the comments into consideration, we have thoroughly revised the manuscript and re-submit the revised manuscript together with the responses to relevant comments. All changes were marked in red in the manuscript and have been approved by all authors.

We hope that the revision is adequate and thank you for considering our manuscript.

If you have any question about this manuscript, please let us know as soon as possible.

Thank you and best wishes.

Yours sincerely,

Ping Mo

College of life and environmental sciences, Hunan University of Arts and Science, Hunan Changde 415000.

E-mail: moping2015@126.com
